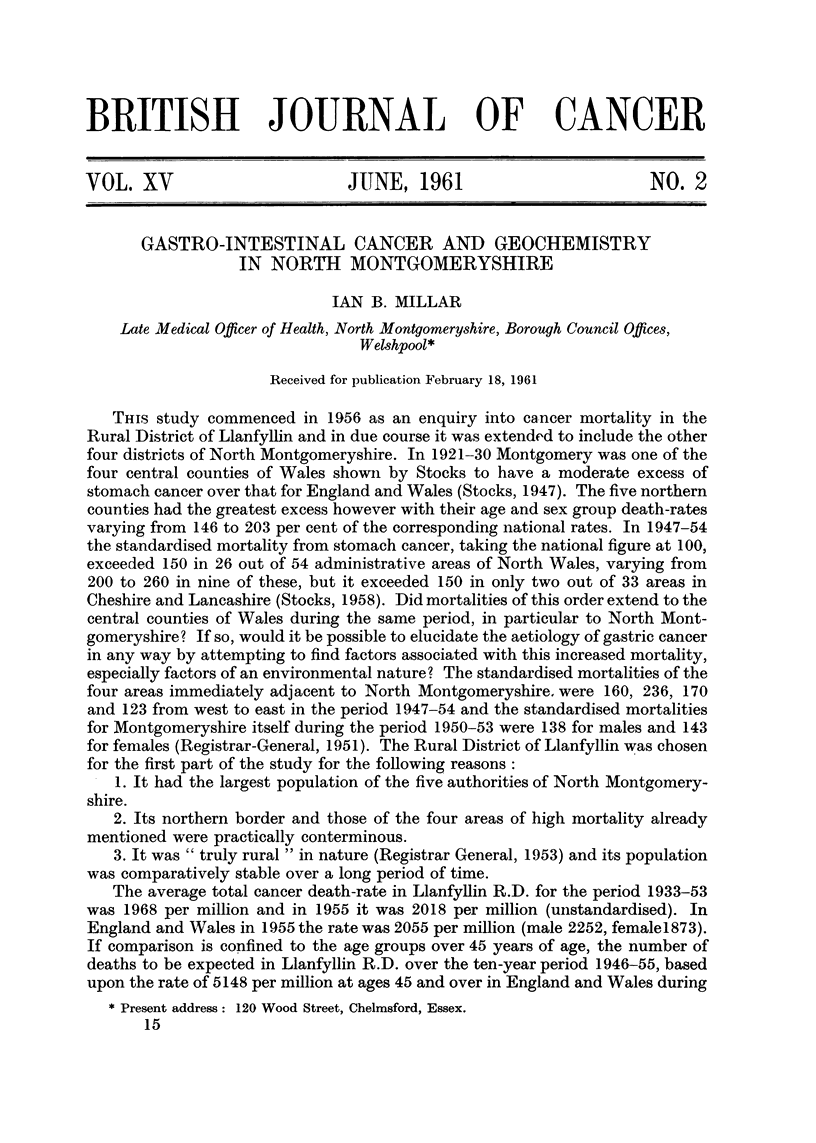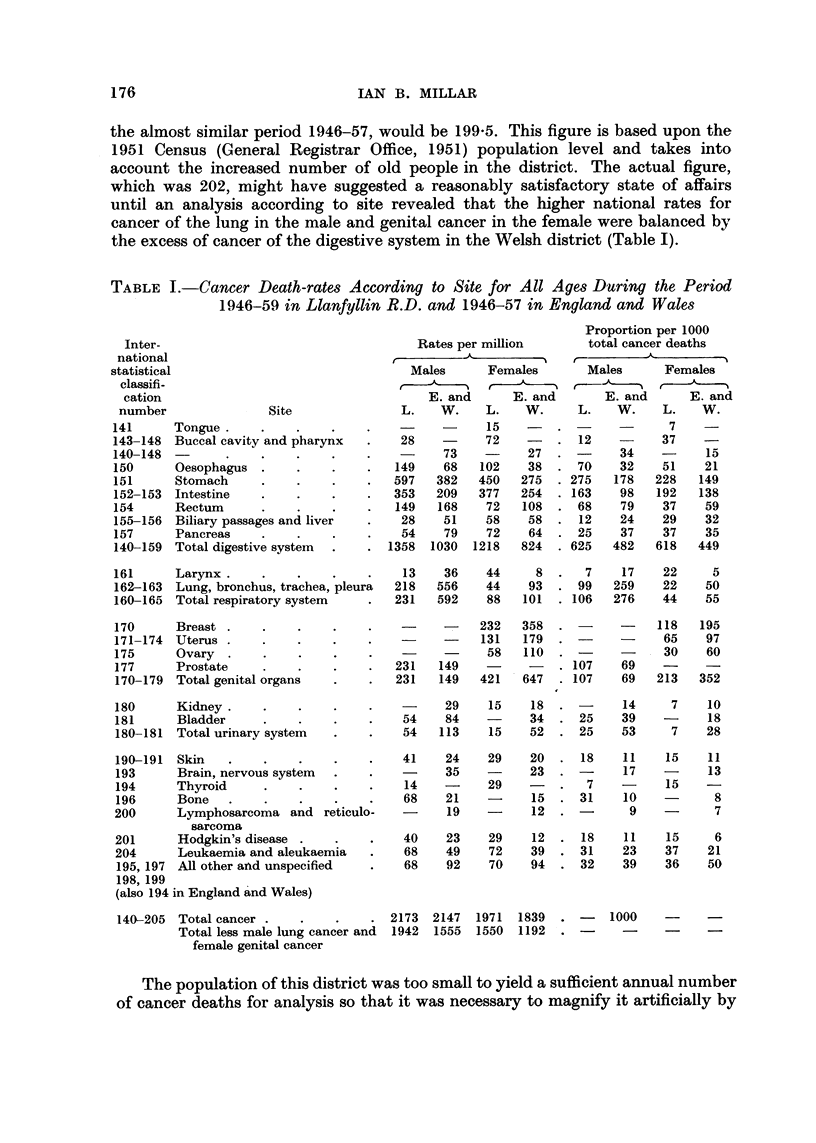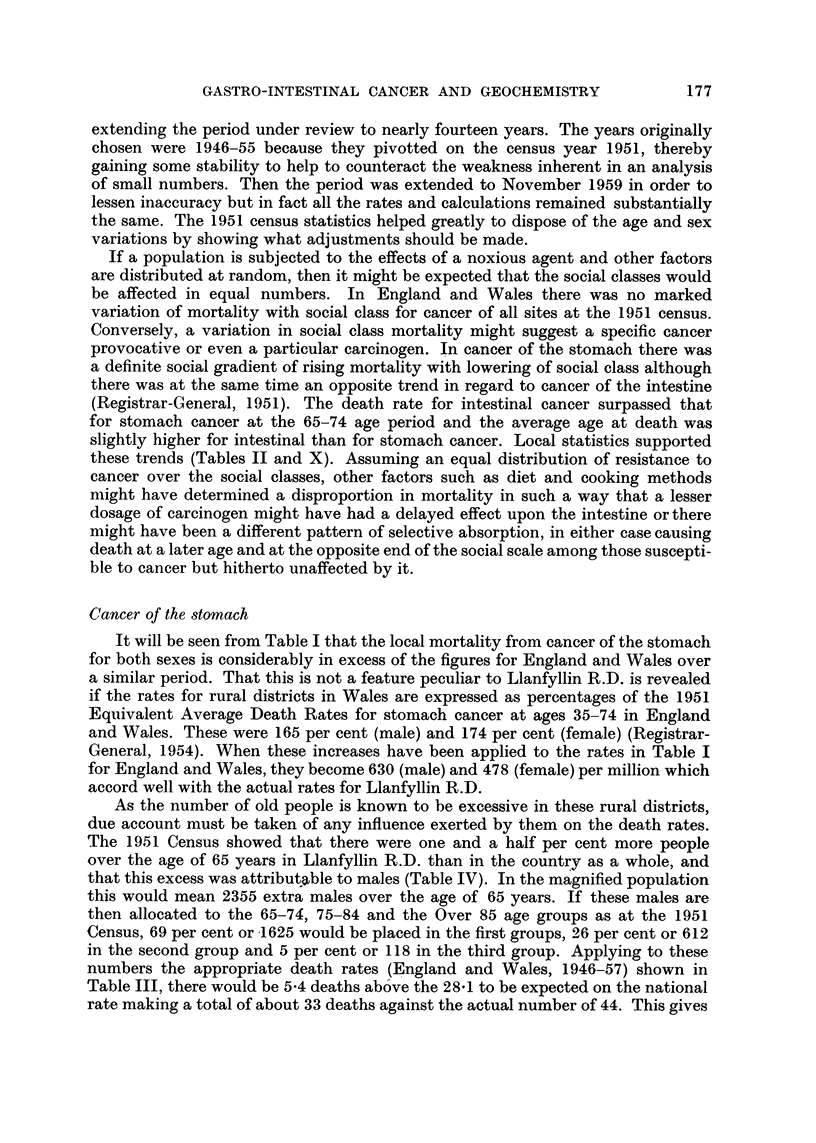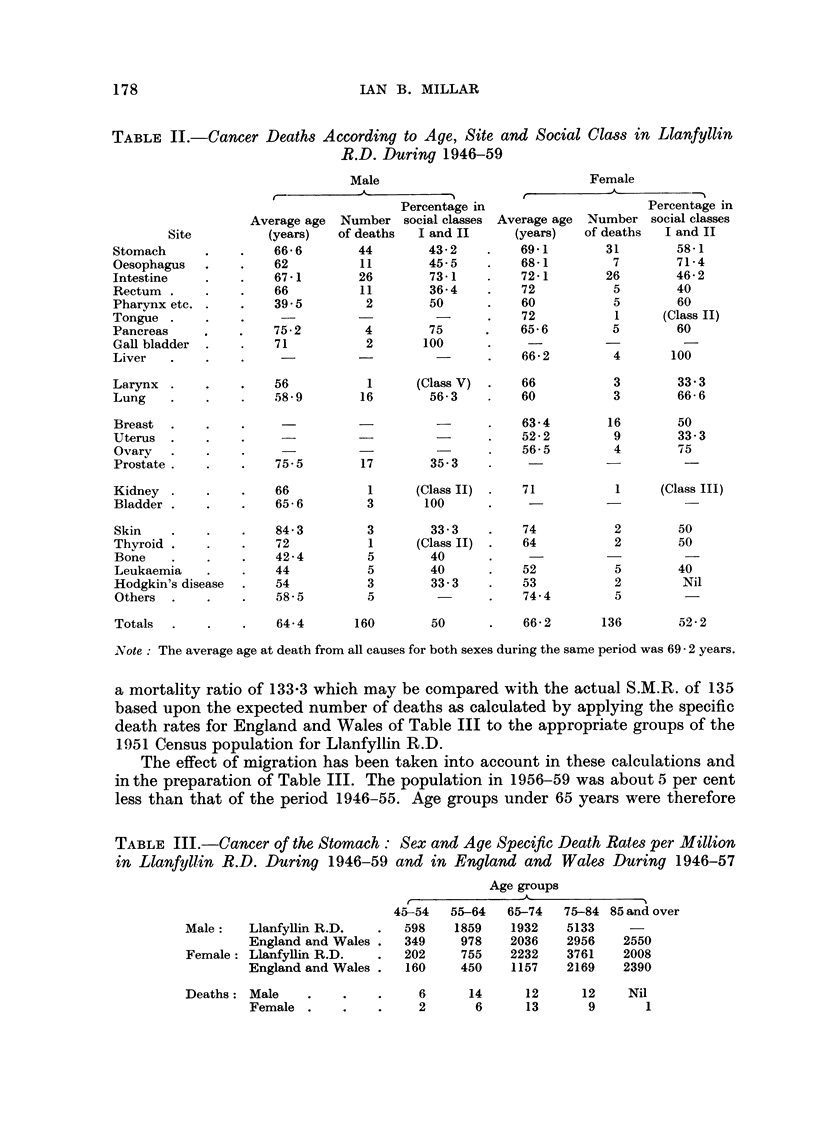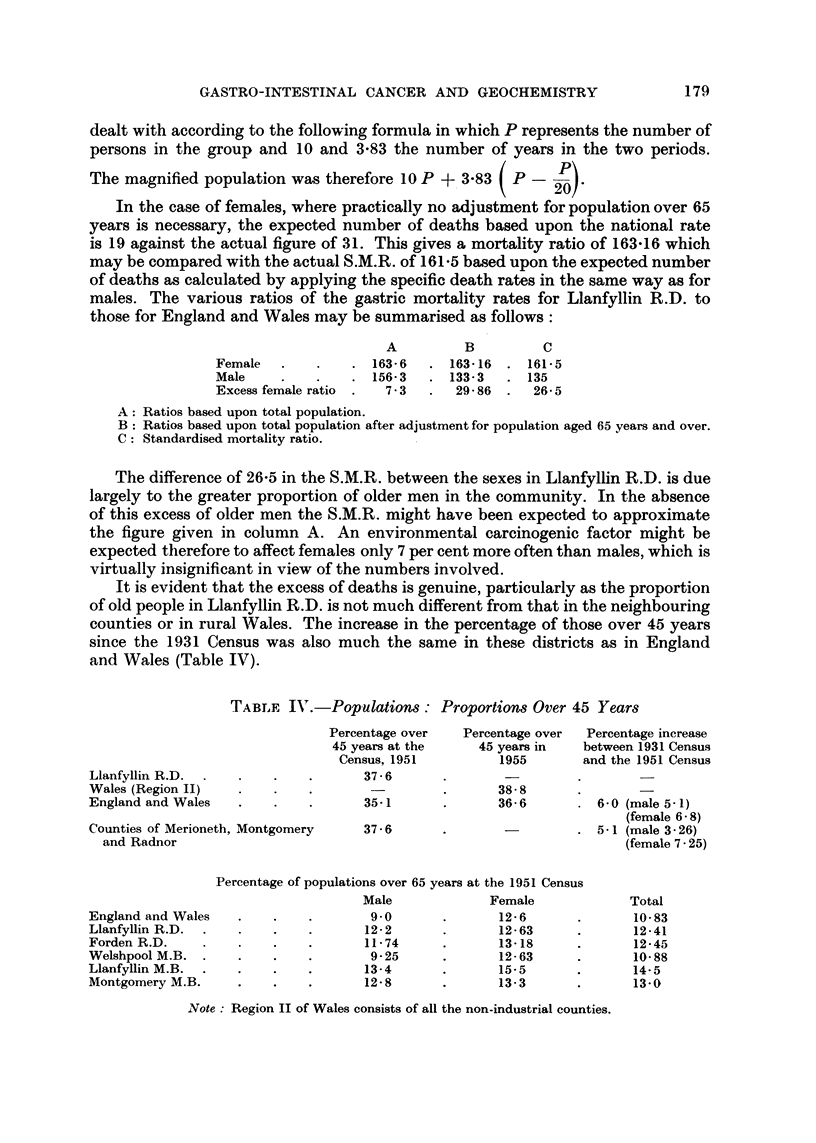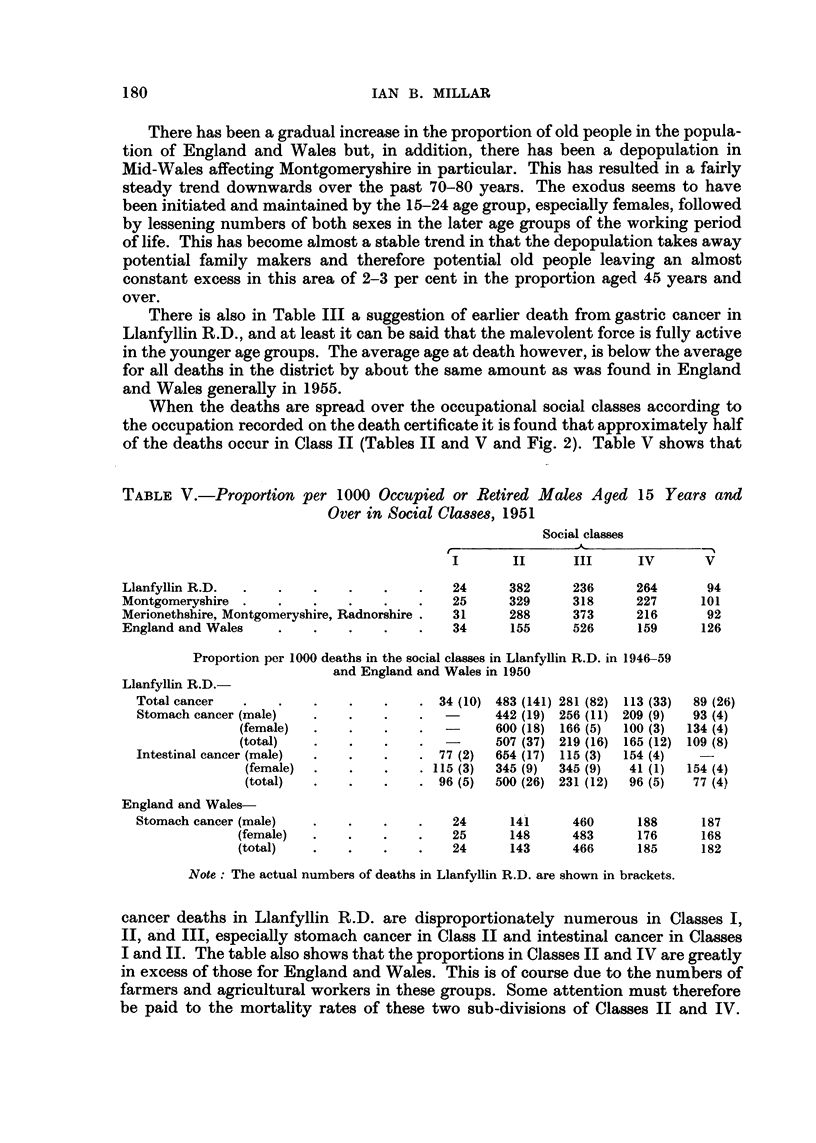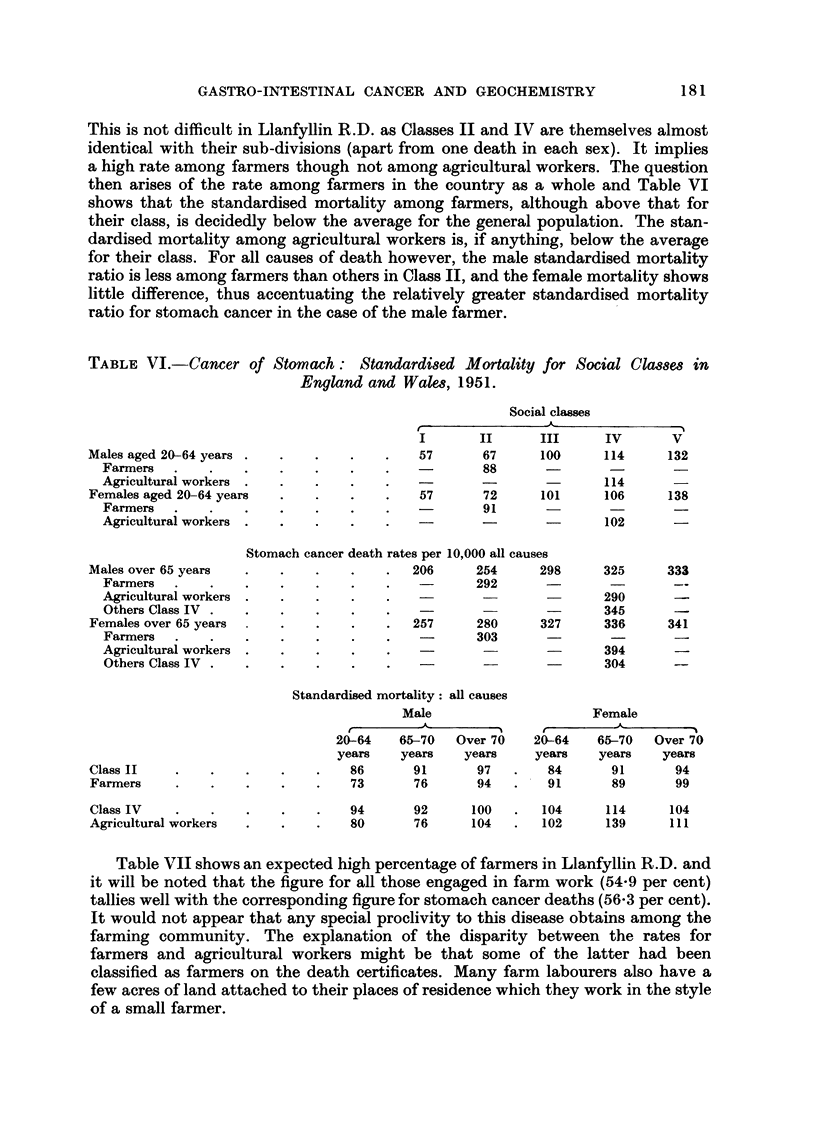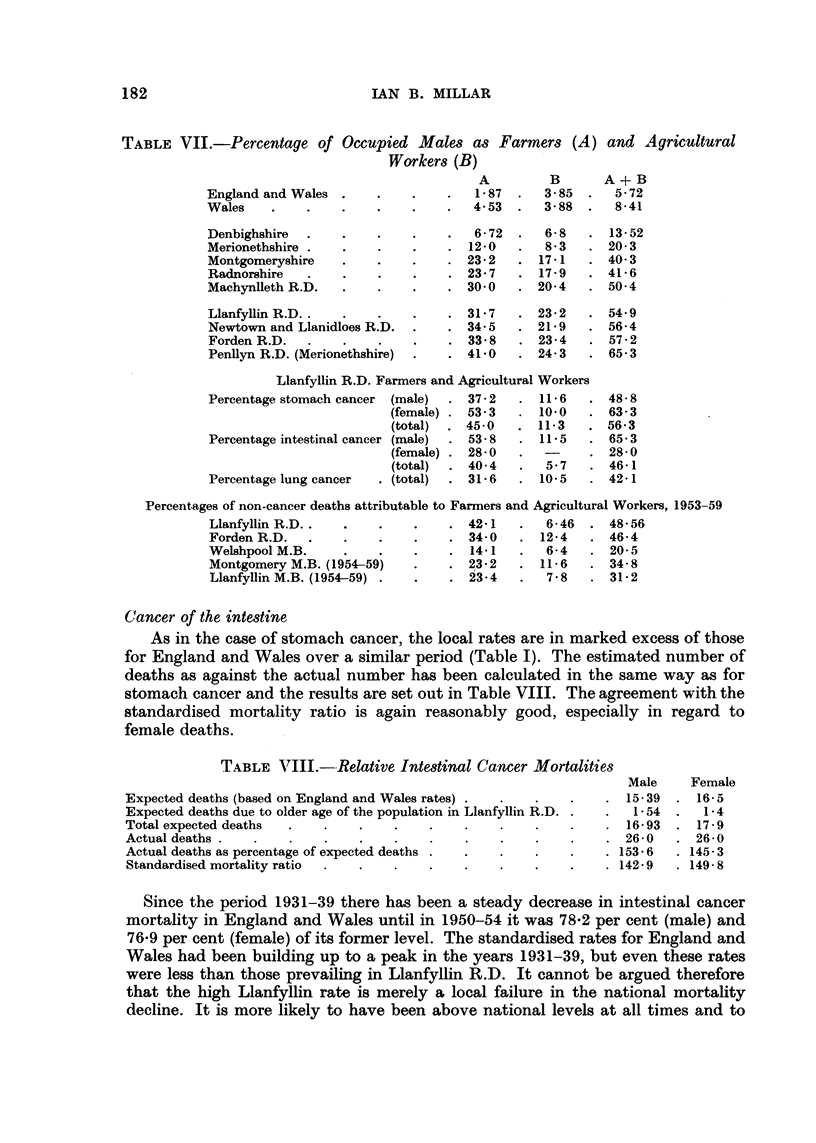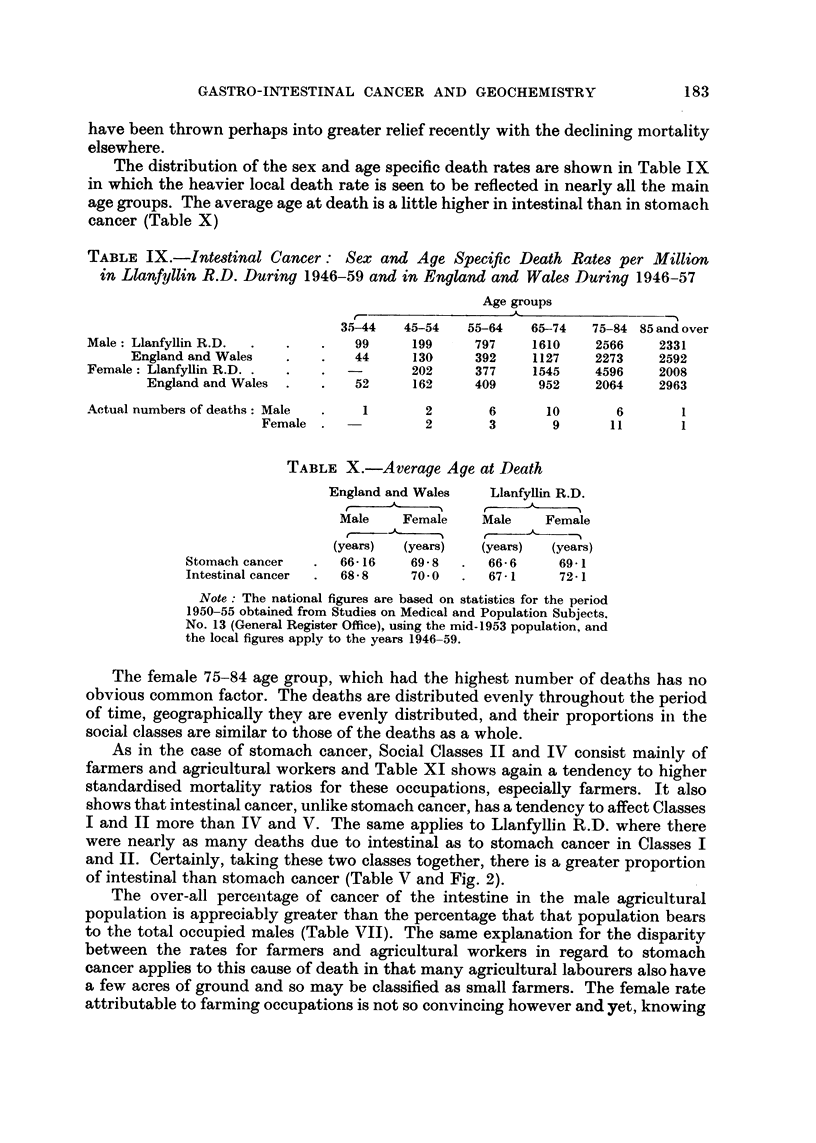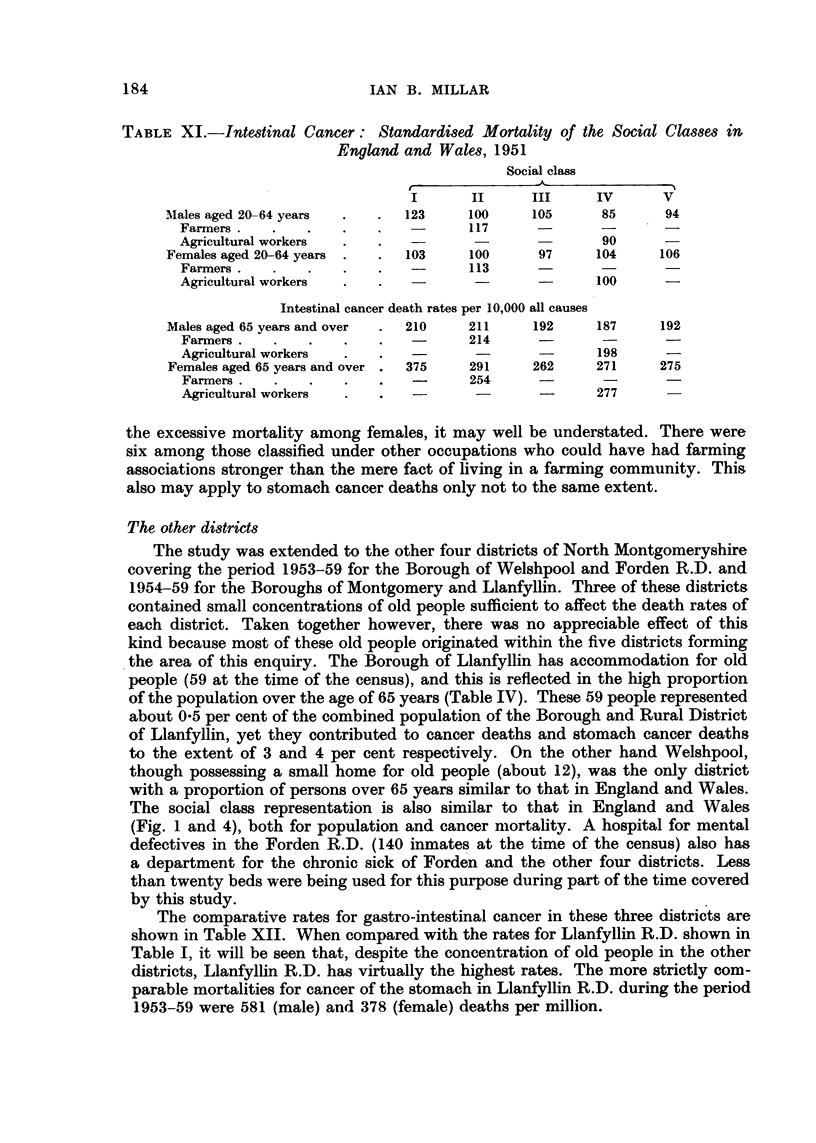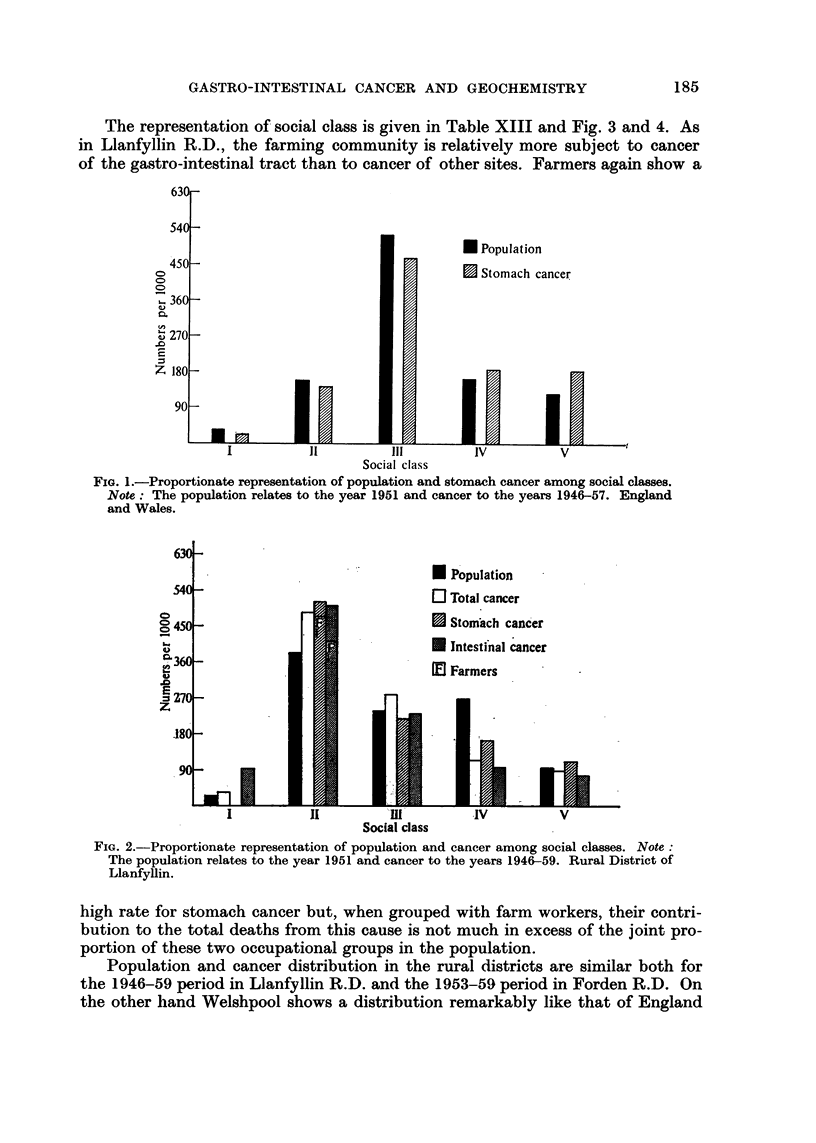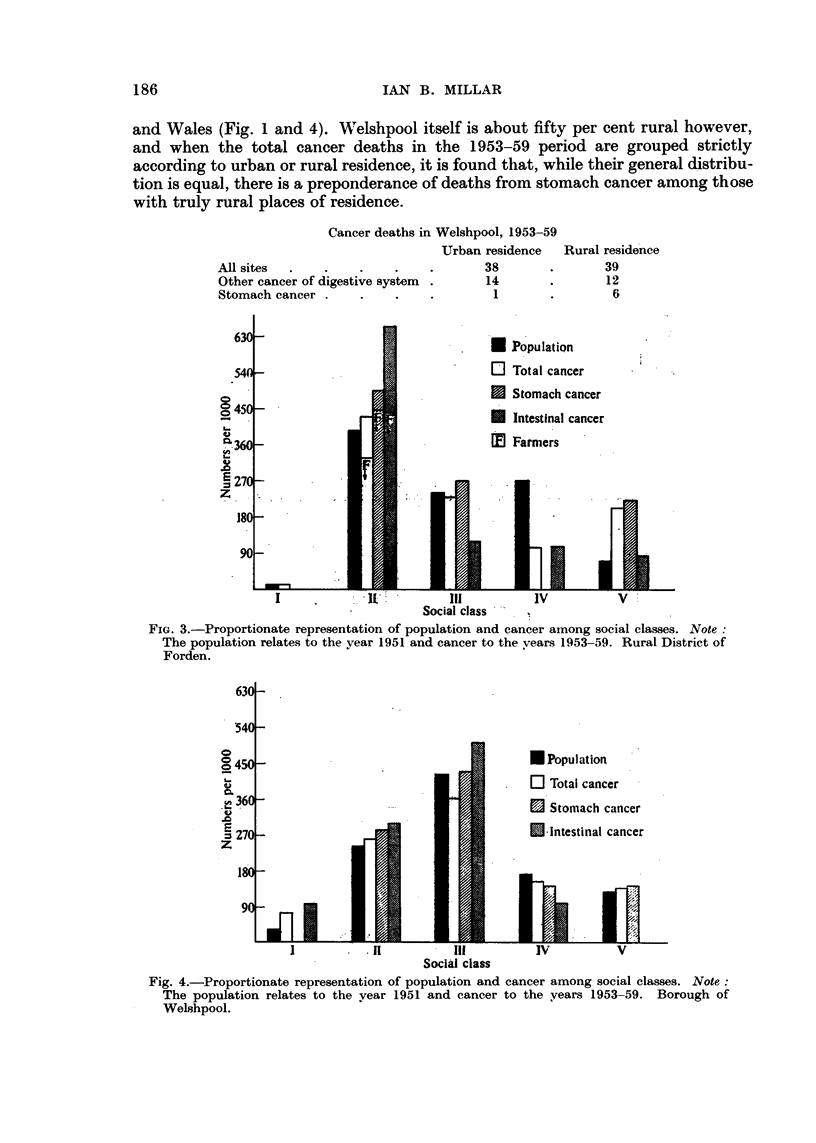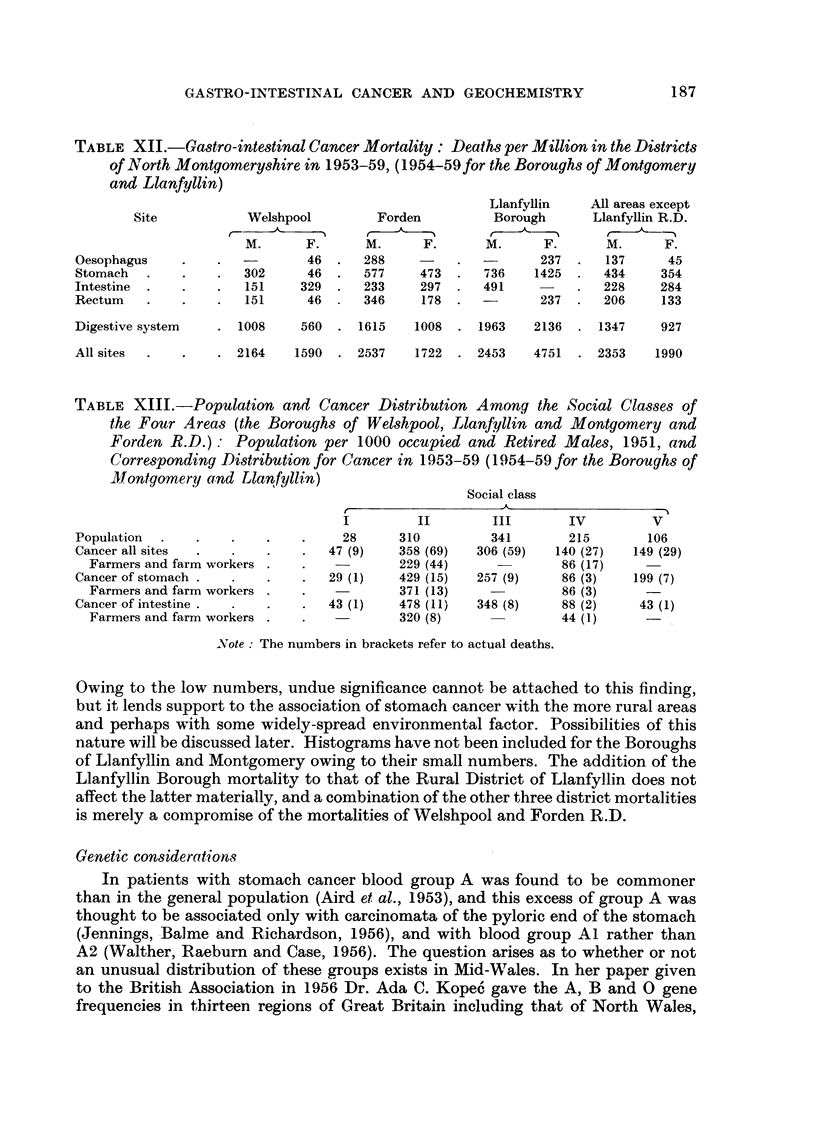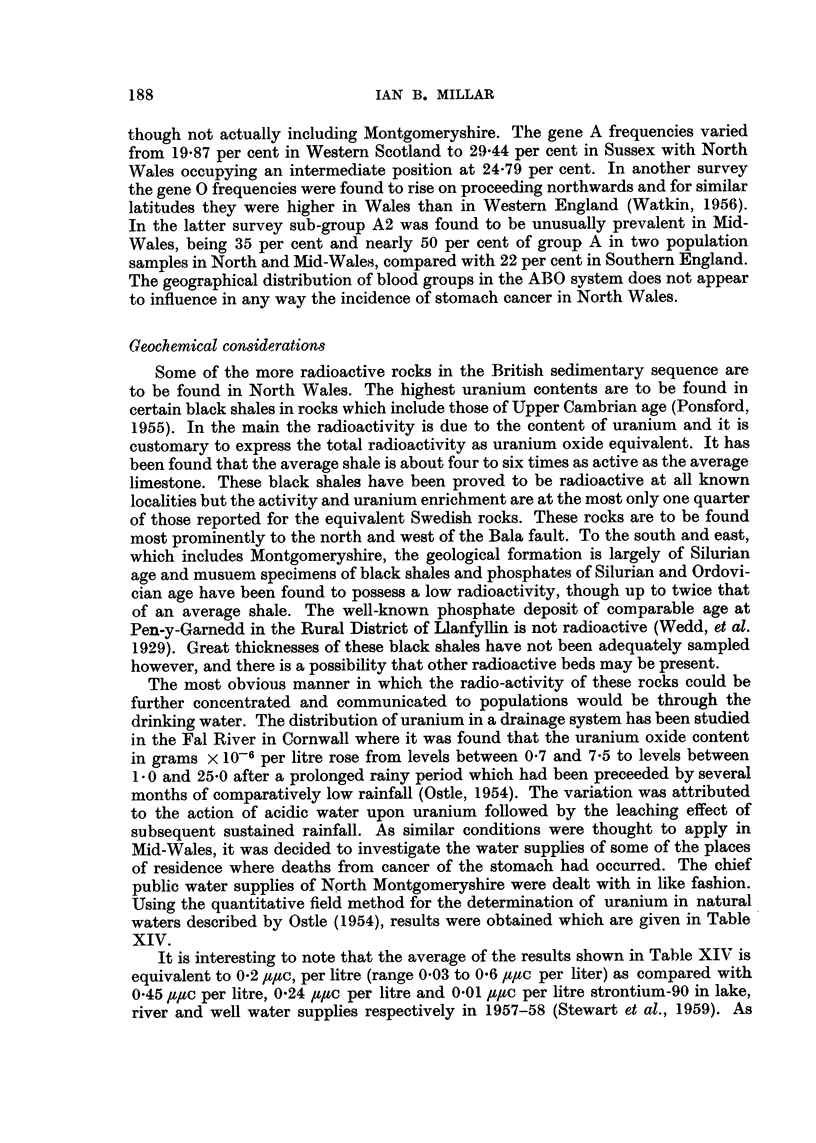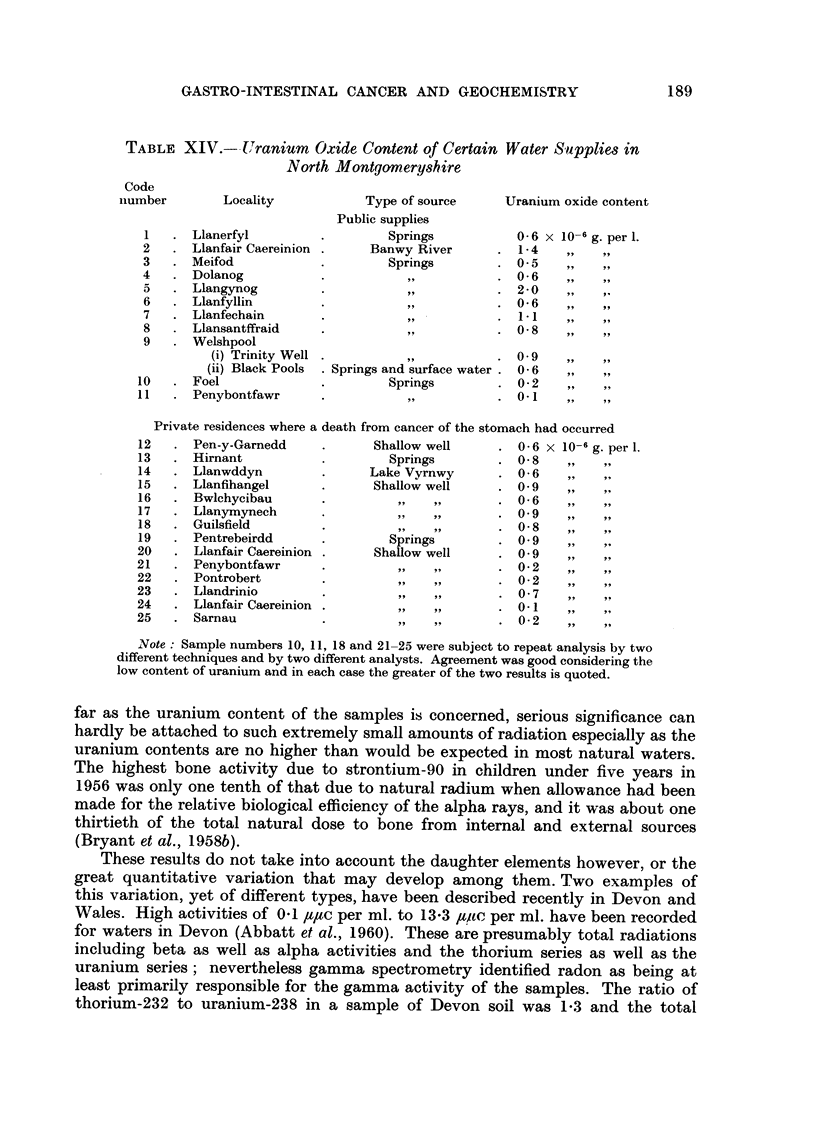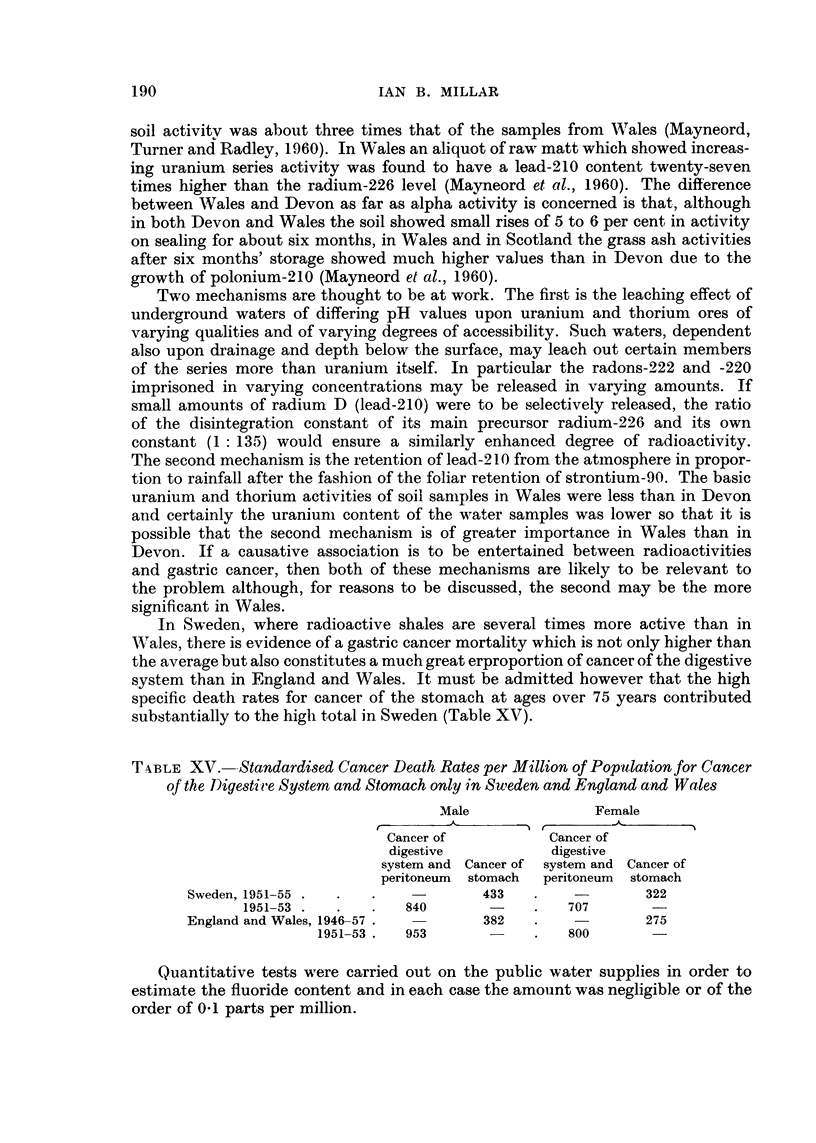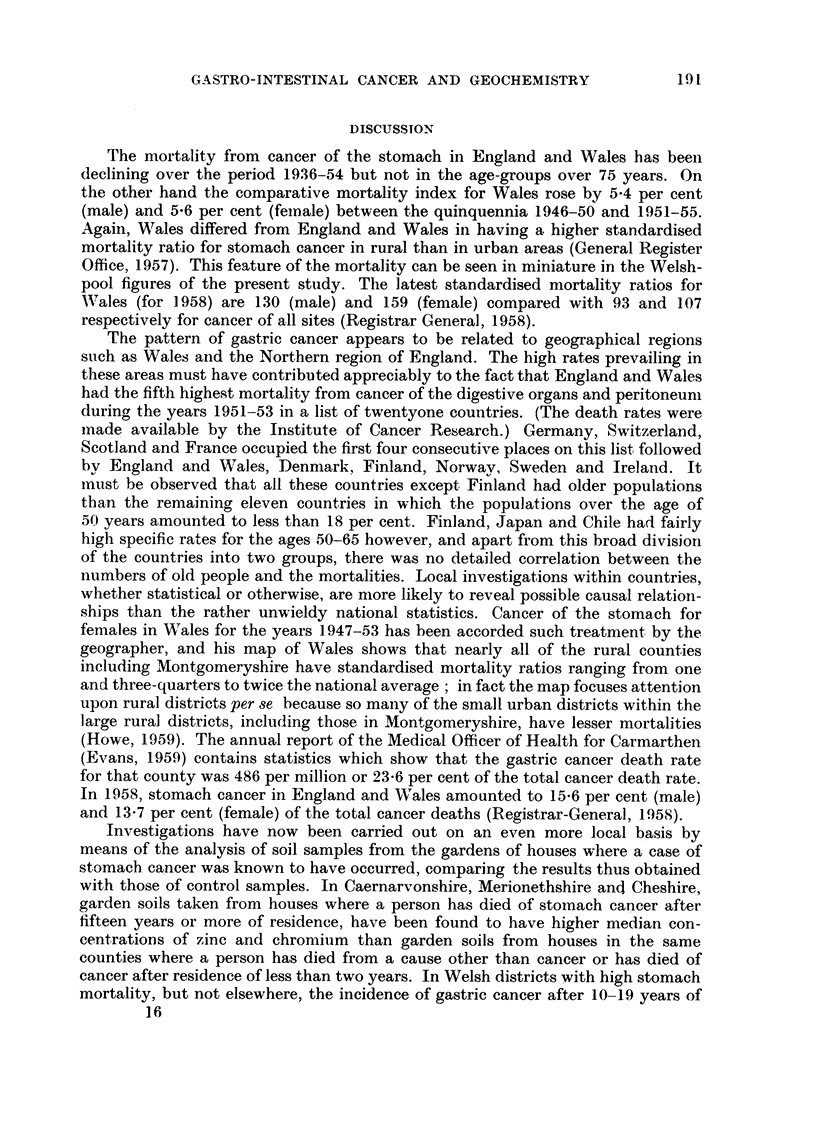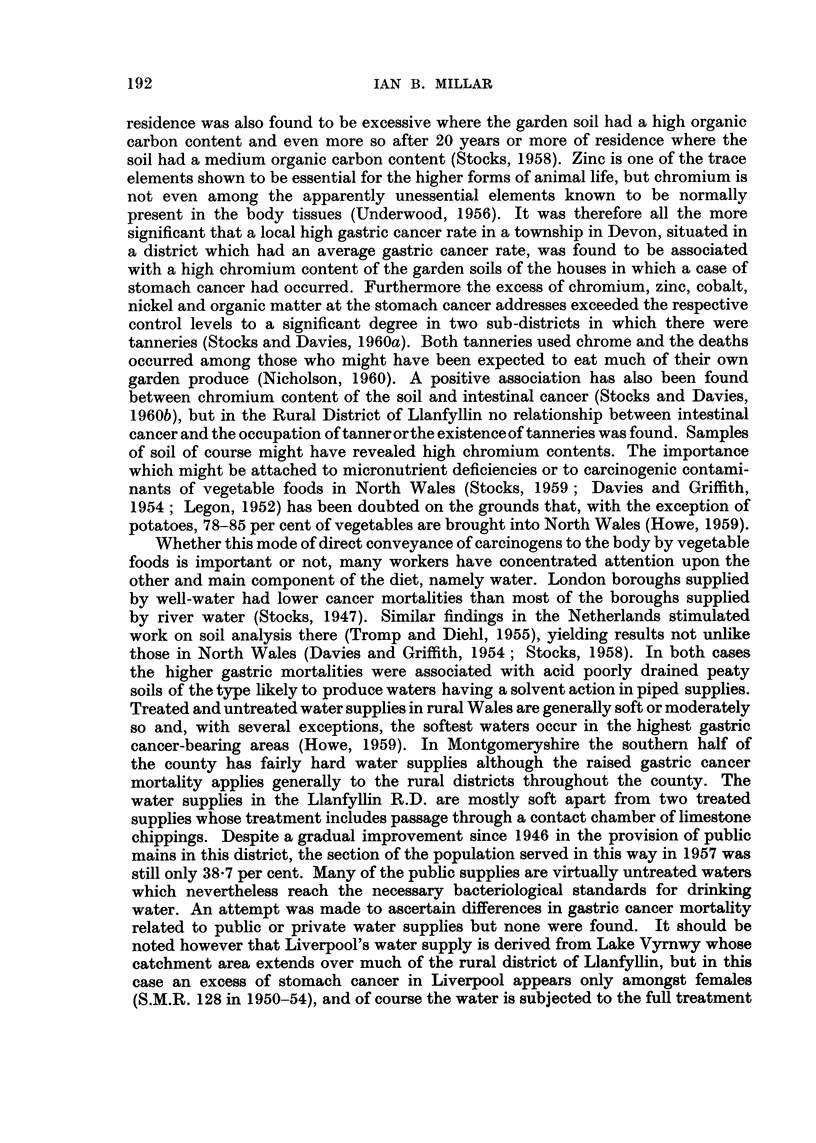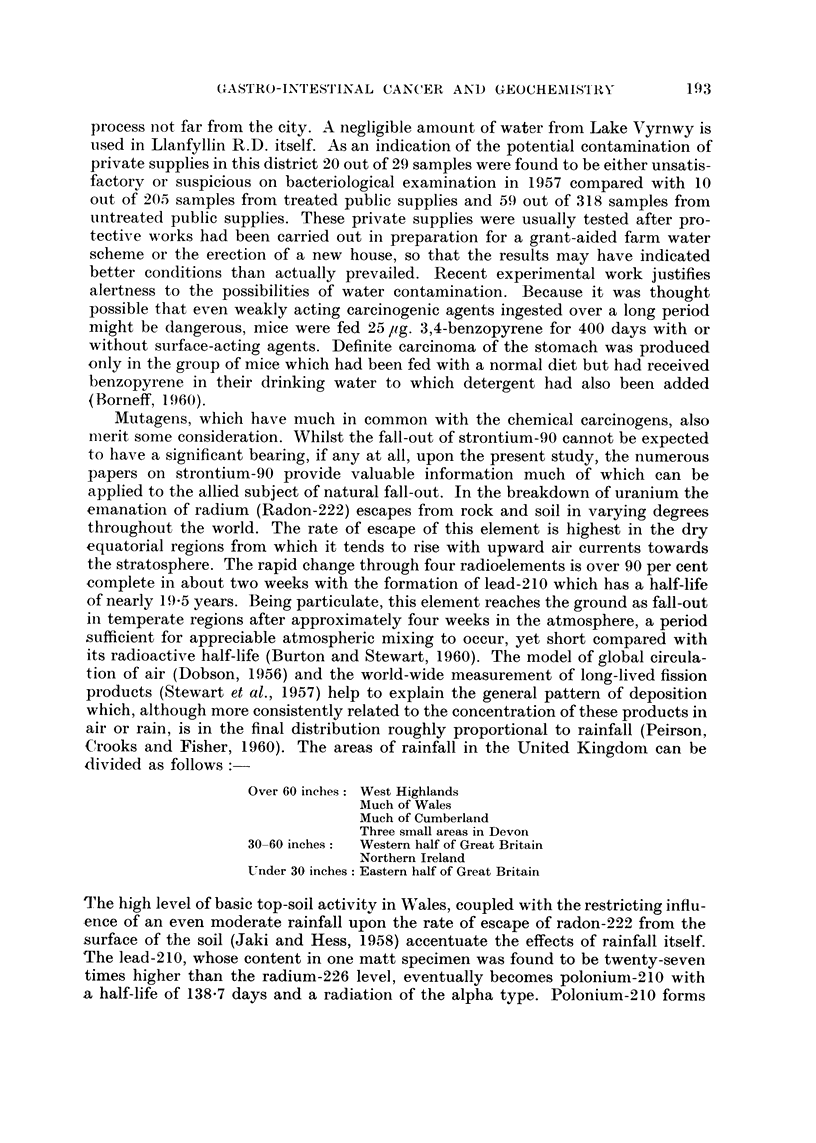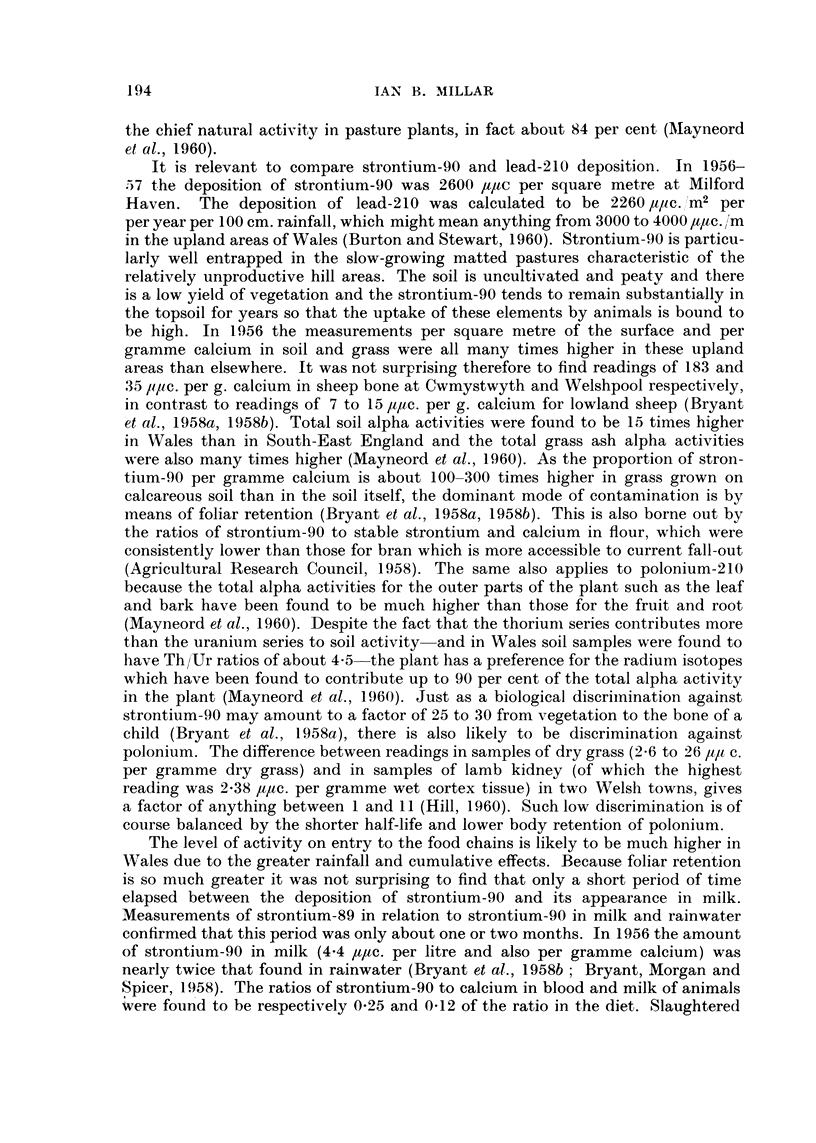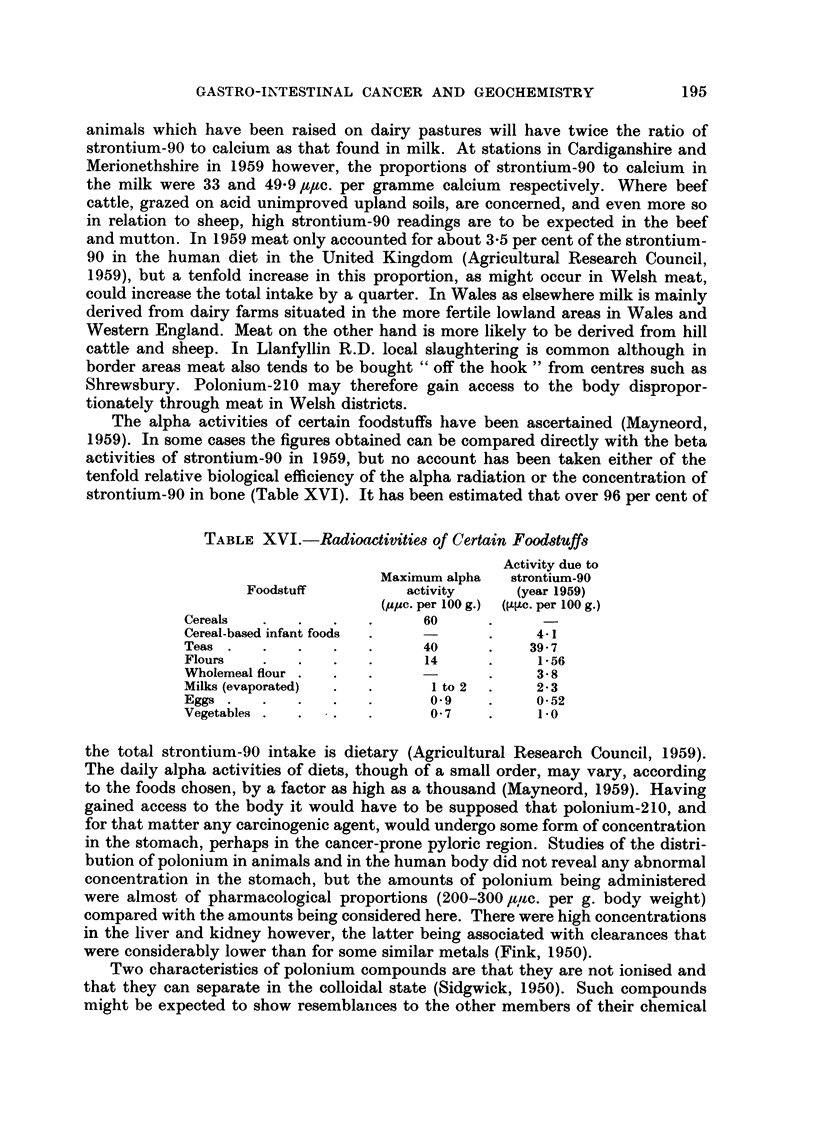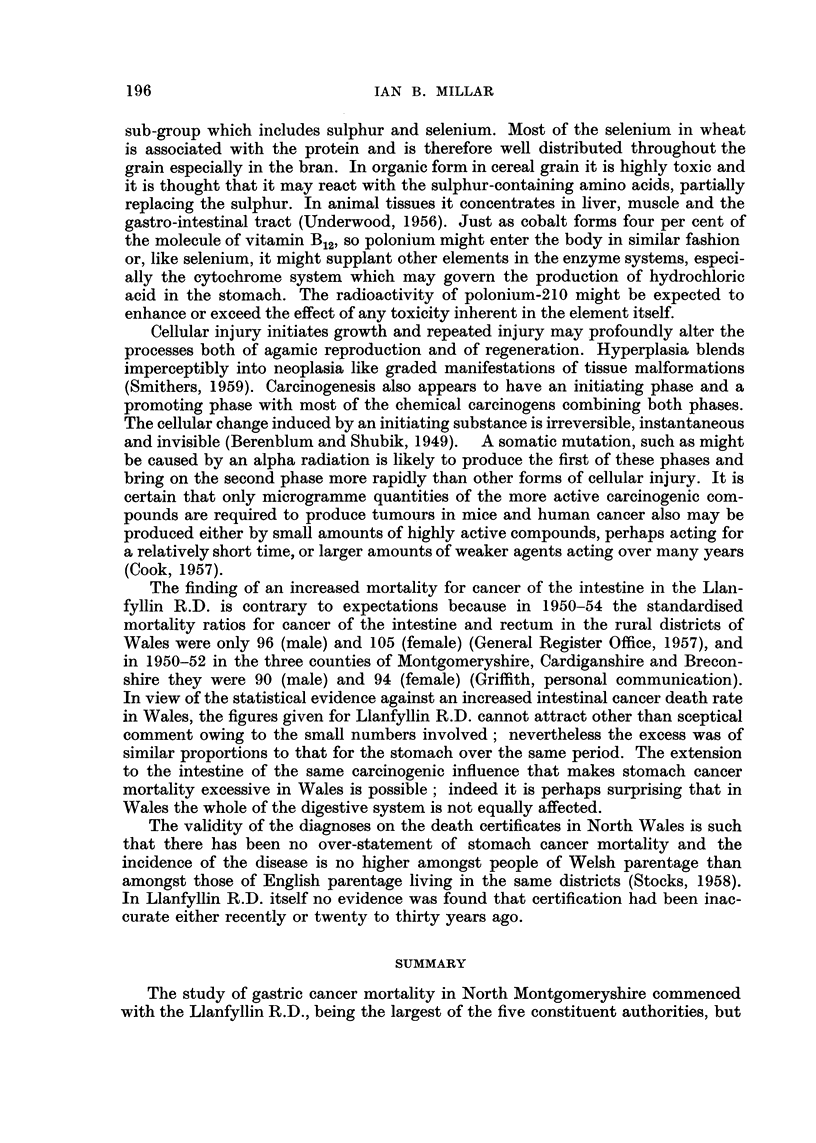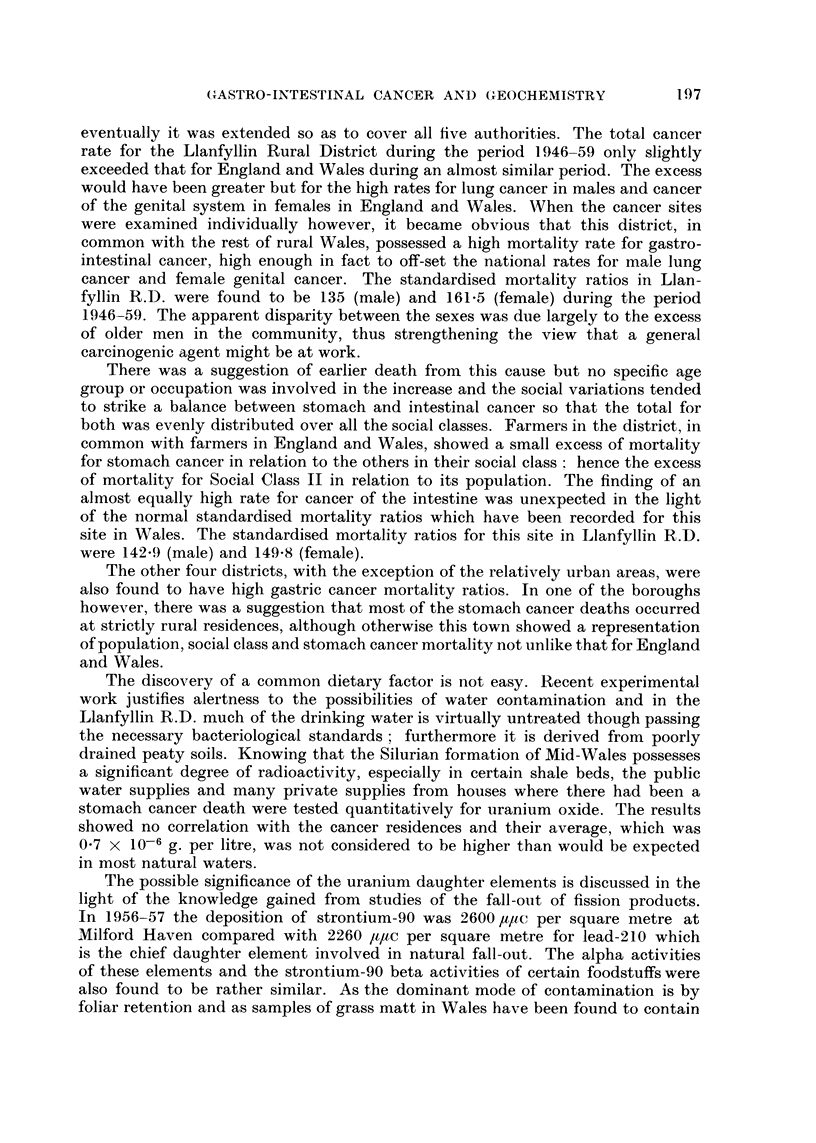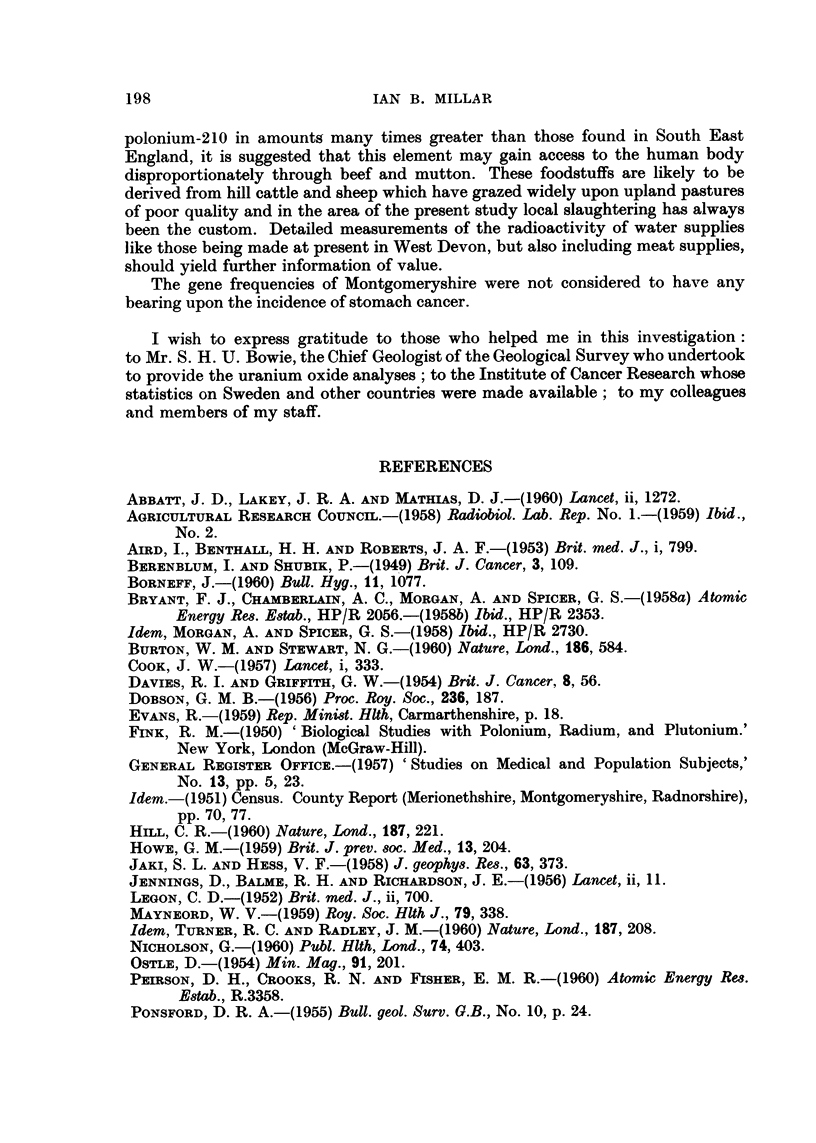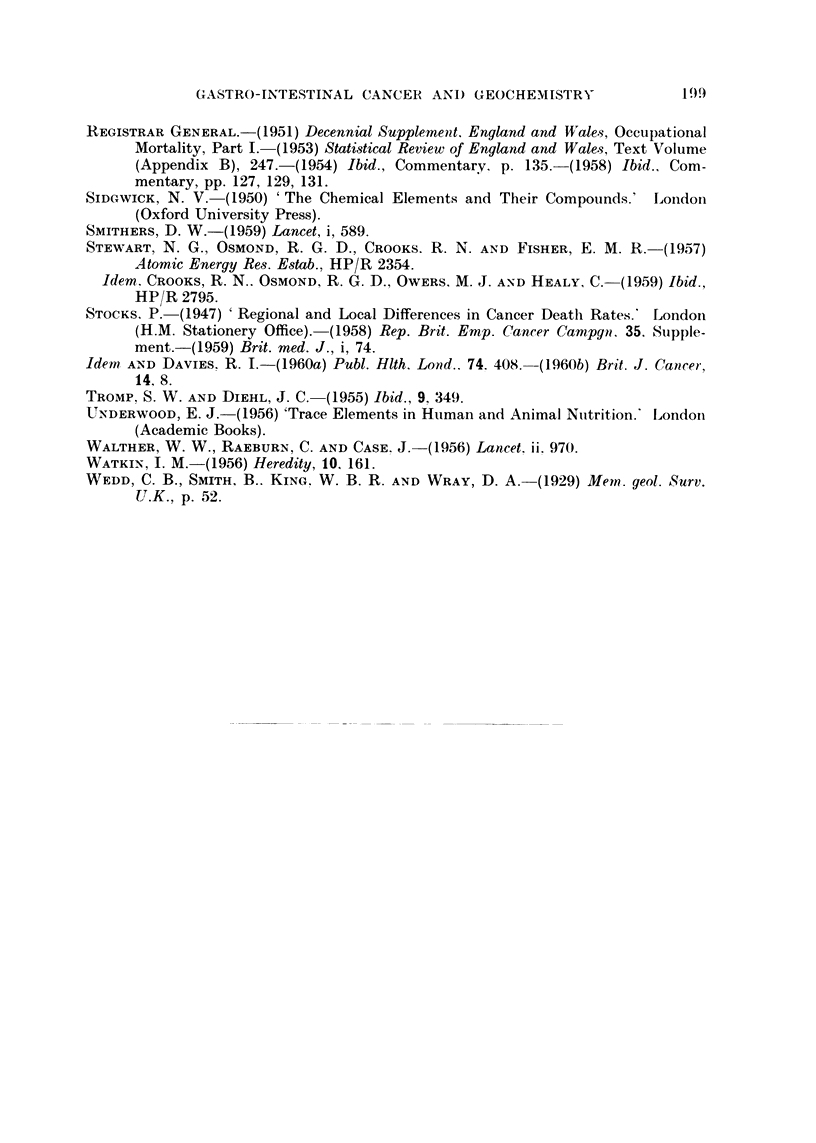# Gastro-intestinal Cancer and Geochemistry in North Montgomeryshire

**DOI:** 10.1038/bjc.1961.23

**Published:** 1961-06

**Authors:** Ian B. Millar


### BRITISH JOURNAL OF CANCER

VOL. XV JUNE, 1961 NO. 2

### GASTRO-INTESTINAL CANCER AND GEOCHEMISTRY IN NORTH MONTGOMERYSHIRE

IAN B. MILLAR

*Late Medical Officer of Health, North Montgomeryshire, Borough Council Offices, Welshpool**

Received for publication February 18, 1961

THIS study commenced in 1956 as an enquiry into cancer mortality in the Rural District of Llanfyllin and in due course it was extended to include the other four districts of North Montgomeryshire. In 1921–30 Montgomery was one of the four central counties of Wales shown by Stocks to have a moderate excess of stomach cancer over that for England and Wales (Stocks, 1947). The five northern counties had the greatest excess however with their age and sex group death-rates varying from 146 to 203 per cent of the corresponding national rates. In 1947–54 the standardised mortality from stomach cancer, taking the national figure at 100, exceeded 150 in 26 out of 54 administrative areas of North Wales, varying from 200 to 260 in nine of these, but it exceeded 150 in only two out of 33 areas in Cheshire and Lancashire (Stocks, 1958). Did mortalities of this order extend to the central counties of Wales during the same period, in particular to North Montgomeryshire? If so, would it be possible to elucidate the aetiology of gastric cancer in any way by attempting to find factors associated with this increased mortality, especially factors of an environmental nature? The standardised mortalities of the four areas immediately adjacent to North Montgomeryshire. were 160, 236, 170 and 123 from west to east in the period 1947–54 and the standardised mortalities for Montgomeryshire itself during the period 1950–53 were 138 for males and 143 for females (Registrar-General, 1951). The Rural District of Llanfyllin was chosen for the first part of the study for the following reasons :

1. It had the largest population of the five authorities of North Montgomeryshire.
2. Its northern border and those of the four areas of high mortality already mentioned were practically conterminous.
3. It was " truly rural " in nature (Registrar General, 1953) and its population was comparatively stable over a long period of time.

The average total cancer death-rate in Llanfyllin R.D. for the period 1933–53 was 1968 per million and in 1955 it was 2018 per million (unstandardised). In England and Wales in 1955 the rate was 2055 per million (male 2252, female1873). If comparison is confined to the age groups over 45 years of age, the number of deaths to be expected in Llanfyllin R.D. over the ten-year period 1946–55, based upon the rate of 5148 per million at ages 45 and over in England and Wales during

* Present address : 120 Wood Street, Chelmsford, Essex.

the almost similar period 1946–57, would be 199·5. This figure is based upon the 1951 Census (General Registrar Office, 1951) population level and takes into account the increased number of old people in the district. The actual figure, which was 202, might have suggested a reasonably satisfactory state of affairs until an analysis according to site revealed that the higher national rates for cancer of the lung in the male and genital cancer in the female were balanced by the excess of cancer of the digestive system in the Welsh district (Table I).

TABLE I.—*Cancer Death-rates According to Site for All Ages During the Period 1946–59 in Llanfyllin R.D. and 1946–57 in England and Wales*

| International statistical classification number | Site | Rates per million | | | | Proportion per 1000 total cancer deaths | | | |
|---|---|---|---|---|---|---|---|---|---|
| | | Males | | Females | | Males | | Females | |
| | | L. | E. and W. | L. | E. and W. | L. | E. and W. | L. | E. and W. |
| 141 | Tongue | — | — | 15 | — | — | — | 7 | — |
| 143–148 | Buccal cavity and pharynx | 28 | — | 72 | — | 12 | — | 37 | — |
| 140–148 | — | — | 73 | — | 27 | — | 34 | — | 15 |
| 150 | Oesophagus | 149 | 68 | 102 | 38 | 70 | 32 | 51 | 21 |
| 151 | Stomach | 597 | 382 | 450 | 275 | 275 | 178 | 228 | 149 |
| 152–153 | Intestine | 353 | 209 | 377 | 254 | 163 | 98 | 192 | 138 |
| 154 | Rectum | 149 | 168 | 72 | 108 | 68 | 79 | 37 | 59 |
| 155–156 | Biliary passages and liver | 28 | 51 | 58 | 58 | 12 | 24 | 29 | 32 |
| 157 | Pancreas | 54 | 79 | 72 | 64 | 25 | 37 | 37 | 35 |
| 140–159 | Total digestive system | 1358 | 1030 | 1218 | 824 | 625 | 482 | 618 | 449 |
| 161 | Larynx | 13 | 36 | 44 | 8 | 7 | 17 | 22 | 5 |
| 162–163 | Lung, bronchus, trachea, pleura | 218 | 556 | 44 | 93 | 99 | 259 | 22 | 50 |
| 160–165 | Total respiratory system | 231 | 592 | 88 | 101 | 106 | 276 | 44 | 55 |
| 170 | Breast | — | — | 232 | 358 | — | — | 118 | 195 |
| 171–174 | Uterus | — | — | 131 | 179 | — | — | 65 | 97 |
| 175 | Ovary | — | — | 58 | 110 | — | — | 30 | 60 |
| 177 | Prostate | 231 | 149 | — | — | 107 | 69 | — | — |
| 170–179 | Total genital organs | 231 | 149 | 421 | 647 | 107 | 69 | 213 | 352 |
| 180 | Kidney | — | 29 | 15 | 18 | — | 14 | 7 | 10 |
| 181 | Bladder | 54 | 84 | — | 34 | 25 | 39 | — | 18 |
| 180–181 | Total urinary system | 54 | 113 | 15 | 52 | 25 | 53 | 7 | 28 |
| 190–191 | Skin | 41 | 24 | 29 | 20 | 18 | 11 | 15 | 11 |
| 193 | Brain, nervous system | — | 35 | — | 23 | — | 17 | — | 13 |
| 194 | Thyroid | 14 | — | 29 | — | 7 | — | 15 | — |
| 196 | Bone | 68 | 21 | — | 15 | 31 | 10 | — | 8 |
| 200 | Lymphosarcoma and reticulosarcoma | — | 19 | — | 12 | — | 9 | — | 7 |
| 201 | Hodgkin's disease | 40 | 23 | 29 | 12 | 18 | 11 | 15 | 6 |
| 204 | Leukaemia and aleukaemia | 68 | 49 | 72 | 39 | 31 | 23 | 37 | 21 |
| 195, 197 198, 199 (also 194 in England and Wales) | All other and unspecified | 68 | 92 | 70 | 94 | 32 | 39 | 36 | 50 |
| 140–205 | Total cancer | 2173 | 2147 | 1971 | 1839 | — | 1000 | — | — |
| | Total less male lung cancer and female genital cancer | 1942 | 1555 | 1550 | 1192 | — | — | — | — |

The population of this district was too small to yield a sufficient annual number of cancer deaths for analysis so that it was necessary to magnify it artificially by extending the period under review to nearly fourteen years. The years originally chosen were 1946–55 because they pivotted on the census year 1951, thereby gaining some stability to help to counteract the weakness inherent in an analysis of small numbers. Then the period was extended to November 1959 in order to lessen inaccuracy but in fact all the rates and calculations remained substantially the same. The 1951 census statistics helped greatly to dispose of the age and sex variations by showing what adjustments should be made.

If a population is subjected to the effects of a noxious agent and other factors are distributed at random, then it might be expected that the social classes would be affected in equal numbers. In England and Wales there was no marked variation of mortality with social class for cancer of all sites at the 1951 census. Conversely, a variation in social class mortality might suggest a specific cancer provocative or even a particular carcinogen. In cancer of the stomach there was a definite social gradient of rising mortality with lowering of social class although there was at the same time an opposite trend in regard to cancer of the intestine (Registrar-General, 1951). The death rate for intestinal cancer surpassed that for stomach cancer at the 65–74 age period and the average age at death was slightly higher for intestinal than for stomach cancer. Local statistics supported these trends (Tables II and X). Assuming an equal distribution of resistance to cancer over the social classes, other factors such as diet and cooking methods might have determined a disproportion in mortality in such a way that a lesser dosage of carcinogen might have had a delayed effect upon the intestine or there might have been a different pattern of selective absorption, in either case causing death at a later age and at the opposite end of the social scale among those susceptible to cancer but hitherto unaffected by it.

*Cancer of the stomach*

It will be seen from Table I that the local mortality from cancer of the stomach for both sexes is considerably in excess of the figures for England and Wales over a similar period. That this is not a feature peculiar to Llanfyllin R.D. is revealed if the rates for rural districts in Wales are expressed as percentages of the 1951 Equivalent Average Death Rates for stomach cancer at ages 35–74 in England and Wales. These were 165 per cent (male) and 174 per cent (female) (Registrar-General, 1954). When these increases have been applied to the rates in Table I for England and Wales, they become 630 (male) and 478 (female) per million which accord well with the actual rates for Llanfyllin R.D.

As the number of old people is known to be excessive in these rural districts, due account must be taken of any influence exerted by them on the death rates. The 1951 Census showed that there were one and a half per cent more people over the age of 65 years in Llanfyllin R.D. than in the country as a whole, and that this excess was attributable to males (Table IV). In the magnified population this would mean 2355 extra males over the age of 65 years. If these males are then allocated to the 65–74, 75–84 and the Over 85 age groups as at the 1951 Census, 69 per cent or 1625 would be placed in the first groups, 26 per cent or 612 in the second group and 5 per cent or 118 in the third group. Applying to these numbers the appropriate death rates (England and Wales, 1946–57) shown in Table III, there would be 5·4 deaths above the 28·1 to be expected on the national rate making a total of about 33 deaths against the actual number of 44. This gives

TABLE II.—*Cancer Deaths According to Age, Site and Social Class in Llanfyllin R.D. During* 1946–59

| Site | Male: Average age (years) | Male: Number of deaths | Male: Percentage in social classes I and II | Female: Average age (years) | Female: Number of deaths | Female: Percentage in social classes I and II |
|---|---|---|---|---|---|---|
| Stomach | 66·6 | 44 | 43·2 | 69·1 | 31 | 58·1 |
| Oesophagus | 62 | 11 | 45·5 | 68·1 | 7 | 71·4 |
| Intestine | 67·1 | 26 | 73·1 | 72·1 | 26 | 46·2 |
| Rectum | 66 | 11 | 36·4 | 72 | 5 | 40 |
| Pharynx etc. | 39·5 | 2 | 50 | 60 | 5 | 60 |
| Tongue | — | — | — | 72 | 1 | (Class II) |
| Pancreas | 75·2 | 4 | 75 | 65·6 | 5 | 60 |
| Gall bladder | 71 | 2 | 100 | — | — | — |
| Liver | — | — | — | 66·2 | 4 | 100 |
| Larynx | 56 | 1 | (Class V) | 66 | 3 | 33·3 |
| Lung | 58·9 | 16 | 56·3 | 60 | 3 | 66·6 |
| Breast | — | — | — | 63·4 | 16 | 50 |
| Uterus | — | — | — | 52·2 | 9 | 33·3 |
| Ovary | — | — | — | 56·5 | 4 | 75 |
| Prostate | 75·5 | 17 | 35·3 | — | — | — |
| Kidney | 66 | 1 | (Class II) | 71 | 1 | (Class III) |
| Bladder | 65·6 | 3 | 100 | — | — | — |
| Skin | 84·3 | 3 | 33·3 | 74 | 2 | 50 |
| Thyroid | 72 | 1 | (Class II) | 64 | 2 | 50 |
| Bone | 42·4 | 5 | 40 | — | — | — |
| Leukaemia | 44 | 5 | 40 | 52 | 5 | 40 |
| Hodgkin's disease | 54 | 3 | 33·3 | 53 | 2 | Nil |
| Others | 58·5 | 5 | — | 74·4 | 5 | — |
| Totals | 64·4 | 160 | 50 | 66·2 | 136 | 52·2 |

*Note :* The average age at death from all causes for both sexes during the same period was 69·2 years.

a mortality ratio of 133·3 which may be compared with the actual S.M.R. of 135 based upon the expected number of deaths as calculated by applying the specific death rates for England and Wales of Table III to the appropriate groups of the 1951 Census population for Llanfyllin R.D.

The effect of migration has been taken into account in these calculations and in the preparation of Table III. The population in 1956–59 was about 5 per cent less than that of the period 1946–55. Age groups under 65 years were therefore

TABLE III.—*Cancer of the Stomach : Sex and Age Specific Death Rates per Million in Llanfyllin R.D. During* 1946–59 *and in England and Wales During* 1946–57

| | | Age groups: 45–54 | 55–64 | 65–74 | 75–84 | 85 and over |
|---|---|---|---|---|---|---|
| Male : | Llanfyllin R.D. | 598 | 1859 | 1932 | 5133 | — |
| | England and Wales | 349 | 978 | 2036 | 2956 | 2550 |
| Female : | Llanfyllin R.D. | 202 | 755 | 2232 | 3761 | 2008 |
| | England and Wales | 160 | 450 | 1157 | 2169 | 2390 |
| Deaths : | Male | 6 | 14 | 12 | 12 | Nil |
| | Female | 2 | 6 | 13 | 9 | 1 |

dealt with according to the following formula in which $P$ represents the number of persons in the group and 10 and 3·83 the number of years in the two periods. The magnified population was therefore $10\,P + 3{\cdot}83\left(P - \frac{P}{20}\right)$.

In the case of females, where practically no adjustment for population over 65 years is necessary, the expected number of deaths based upon the national rate is 19 against the actual figure of 31. This gives a mortality ratio of 163·16 which may be compared with the actual S.M.R. of 161·5 based upon the expected number of deaths as calculated by applying the specific death rates in the same way as for males. The various ratios of the gastric mortality rates for Llanfyllin R.D. to those for England and Wales may be summarised as follows :

| | A | B | C |
|---|---|---|---|
| Female | 163·6 | 163·16 | 161·5 |
| Male | 156·3 | 133·3 | 135 |
| Excess female ratio | 7·3 | 29·86 | 26·5 |

A : Ratios based upon total population.
B : Ratios based upon total population after adjustment for population aged 65 years and over.
C : Standardised mortality ratio.

The difference of 26·5 in the S.M.R. between the sexes in Llanfyllin R.D. is due largely to the greater proportion of older men in the community. In the absence of this excess of older men the S.M.R. might have been expected to approximate the figure given in column A. An environmental carcinogenic factor might be expected therefore to affect females only 7 per cent more often than males, which is virtually insignificant in view of the numbers involved.

It is evident that the excess of deaths is genuine, particularly as the proportion of old people in Llanfyllin R.D. is not much different from that in the neighbouring counties or in rural Wales. The increase in the percentage of those over 45 years since the 1931 Census was also much the same in these districts as in England and Wales (Table IV).

TABLE IV.—*Populations : Proportions Over 45 Years*

| | Percentage over 45 years at the Census, 1951 | Percentage over 45 years in 1955 | Percentage increase between 1931 Census and the 1951 Census |
|---|---|---|---|
| Llanfyllin R.D. | 37·6 | — | — |
| Wales (Region II) | — | 38·8 | — |
| England and Wales | 35·1 | 36·6 | 6·0 (male 5·1) (female 6·8) |
| Counties of Merioneth, Montgomery and Radnor | 37·6 | — | 5·1 (male 3·26) (female 7·25) |

Percentage of populations over 65 years at the 1951 Census

| | Male | Female | Total |
|---|---|---|---|
| England and Wales | 9·0 | 12·6 | 10·83 |
| Llanfyllin R.D. | 12·2 | 12·63 | 12·41 |
| Forden R.D. | 11·74 | 13·18 | 12·45 |
| Welshpool M.B. | 9·25 | 12·63 | 10·88 |
| Llanfyllin M.B. | 13·4 | 15·5 | 14·5 |
| Montgomery M.B. | 12·8 | 13·3 | 13·0 |

*Note :* Region II of Wales consists of all the non-industrial counties.

There has been a gradual increase in the proportion of old people in the population of England and Wales but, in addition, there has been a depopulation in Mid-Wales affecting Montgomeryshire in particular. This has resulted in a fairly steady trend downwards over the past 70–80 years. The exodus seems to have been initiated and maintained by the 15–24 age group, especially females, followed by lessening numbers of both sexes in the later age groups of the working period of life. This has become almost a stable trend in that the depopulation takes away potential family makers and therefore potential old people leaving an almost constant excess in this area of 2–3 per cent in the proportion aged 45 years and over.

There is also in Table III a suggestion of earlier death from gastric cancer in Llanfyllin R.D., and at least it can be said that the malevolent force is fully active in the younger age groups. The average age at death however, is below the average for all deaths in the district by about the same amount as was found in England and Wales generally in 1955.

When the deaths are spread over the occupational social classes according to the occupation recorded on the death certificate it is found that approximately half of the deaths occur in Class II (Tables II and V and Fig. 2). Table V shows that

TABLE V.—*Proportion per* 1000 *Occupied or Retired Males Aged* 15 *Years and Over in Social Classes*, 1951

| | Social classes | | | | |
|---|---|---|---|---|---|
| | I | II | III | IV | V |
| Llanfyllin R.D. | 24 | 382 | 236 | 264 | 94 |
| Montgomeryshire | 25 | 329 | 318 | 227 | 101 |
| Merionethshire, Montgomeryshire, Radnorshire | 31 | 288 | 373 | 216 | 92 |
| England and Wales | 34 | 155 | 526 | 159 | 126 |
| *Proportion per 1000 deaths in the social classes in Llanfyllin R.D. in 1946–59 and England and Wales in 1950* | | | | | |
| Llanfyllin R.D.— | | | | | |
| Total cancer | 34 (10) | 483 (141) | 281 (82) | 113 (33) | 89 (26) |
| Stomach cancer (male) | — | 442 (19) | 256 (11) | 209 (9) | 93 (4) |
| (female) | — | 600 (18) | 166 (5) | 100 (3) | 134 (4) |
| (total) | — | 507 (37) | 219 (16) | 165 (12) | 109 (8) |
| Intestinal cancer (male) | 77 (2) | 654 (17) | 115 (3) | 154 (4) | — |
| (female) | 115 (3) | 345 (9) | 345 (9) | 41 (1) | 154 (4) |
| (total) | 96 (5) | 500 (26) | 231 (12) | 96 (5) | 77 (4) |
| England and Wales— | | | | | |
| Stomach cancer (male) | 24 | 141 | 460 | 188 | 187 |
| (female) | 25 | 148 | 483 | 176 | 168 |
| (total) | 24 | 143 | 466 | 185 | 182 |

*Note :* The actual numbers of deaths in Llanfyllin R.D. are shown in brackets.

cancer deaths in Llanfyllin R.D. are disproportionately numerous in Classes I, II, and III, especially stomach cancer in Class II and intestinal cancer in Classes I and II. The table also shows that the proportions in Classes II and IV are greatly in excess of those for England and Wales. This is of course due to the numbers of farmers and agricultural workers in these groups. Some attention must therefore be paid to the mortality rates of these two sub-divisions of Classes II and IV.

This is not difficult in Llanfyllin R.D. as Classes II and IV are themselves almost identical with their sub-divisions (apart from one death in each sex). It implies a high rate among farmers though not among agricultural workers. The question then arises of the rate among farmers in the country as a whole and Table VI shows that the standardised mortality among farmers, although above that for their class, is decidedly below the average for the general population. The standardised mortality among agricultural workers is, if anything, below the average for their class. For all causes of death however, the male standardised mortality ratio is less among farmers than others in Class II, and the female mortality shows little difference, thus accentuating the relatively greater standardised mortality ratio for stomach cancer in the case of the male farmer.

TABLE VI.—*Cancer of Stomach: Standardised Mortality for Social Classes in England and Wales*, 1951.

| | Social classes | | | | |
|---|---|---|---|---|---|
| | I | II | III | IV | V |
| Males aged 20–64 years | 57 | 67 | 100 | 114 | 132 |
| Farmers | — | 88 | — | — | — |
| Agricultural workers | — | — | — | 114 | — |
| Females aged 20–64 years | 57 | 72 | 101 | 106 | 138 |
| Farmers | — | 91 | — | — | — |
| Agricultural workers | — | — | — | 102 | — |
| | Stomach cancer death rates per 10,000 all causes | | | | |
| Males over 65 years | 206 | 254 | 298 | 325 | 333 |
| Farmers | — | 292 | — | — | — |
| Agricultural workers | — | — | — | 290 | — |
| Others Class IV | — | — | — | 345 | — |
| Females over 65 years | 257 | 280 | 327 | 336 | 341 |
| Farmers | — | 303 | — | — | — |
| Agricultural workers | — | — | — | 394 | — |
| Others Class IV | — | — | — | 304 | — |

Standardised mortality: all causes

| | Male | | | Female | | |
|---|---|---|---|---|---|---|
| | 20–64 years | 65–70 years | Over 70 years | 20–64 years | 65–70 years | Over 70 years |
| Class II | 86 | 91 | 97 | 84 | 91 | 94 |
| Farmers | 73 | 76 | 94 | 91 | 89 | 99 |
| Class IV | 94 | 92 | 100 | 104 | 114 | 104 |
| Agricultural workers | 80 | 76 | 104 | 102 | 139 | 111 |

Table VII shows an expected high percentage of farmers in Llanfyllin R.D. and it will be noted that the figure for all those engaged in farm work (54·9 per cent) tallies well with the corresponding figure for stomach cancer deaths (56·3 per cent). It would not appear that any special proclivity to this disease obtains among the farming community. The explanation of the disparity between the rates for farmers and agricultural workers might be that some of the latter had been classified as farmers on the death certificates. Many farm labourers also have a few acres of land attached to their places of residence which they work in the style of a small farmer.

TABLE VII.—*Percentage of Occupied Males as Farmers (A) and Agricultural Workers (B)*

| | | A | B | A + B |
|---|---|---|---|---|
| England and Wales | | 1·87 | 3·85 | 5·72 |
| Wales | | 4·53 | 3·88 | 8·41 |
| Denbighshire | | 6·72 | 6·8 | 13·52 |
| Merionethshire | | 12·0 | 8·3 | 20·3 |
| Montgomeryshire | | 23·2 | 17·1 | 40·3 |
| Radnorshire | | 23·7 | 17·9 | 41·6 |
| Machynlleth R.D. | | 30·0 | 20·4 | 50·4 |
| Llanfyllin R.D. | | 31·7 | 23·2 | 54·9 |
| Newtown and Llanidloes R.D. | | 34·5 | 21·9 | 56·4 |
| Forden R.D. | | 33·8 | 23·4 | 57·2 |
| Penllyn R.D. (Merionethshire) | | 41·0 | 24·3 | 65·3 |
| Llanfyllin R.D. Farmers and Agricultural Workers | | | | |
| Percentage stomach cancer | (male) | 37·2 | 11·6 | 48·8 |
| | (female) | 53·3 | 10·0 | 63·3 |
| | (total) | 45·0 | 11·3 | 56·3 |
| Percentage intestinal cancer | (male) | 53·8 | 11·5 | 65·3 |
| | (female) | 28·0 | — | 28·0 |
| | (total) | 40·4 | 5·7 | 46·1 |
| Percentage lung cancer | (total) | 31·6 | 10·5 | 42·1 |
| Percentages of non-cancer deaths attributable to Farmers and Agricultural Workers, 1953–59 | | | | |
| Llanfyllin R.D. | | 42·1 | 6·46 | 48·56 |
| Forden R.D. | | 34·0 | 12·4 | 46·4 |
| Welshpool M.B. | | 14·1 | 6·4 | 20·5 |
| Montgomery M.B. (1954–59) | | 23·2 | 11·6 | 34·8 |
| Llanfyllin M.B. (1954–59) | | 23·4 | 7·8 | 31·2 |

*Cancer of the intestine*

As in the case of stomach cancer, the local rates are in marked excess of those for England and Wales over a similar period (Table I). The estimated number of deaths as against the actual number has been calculated in the same way as for stomach cancer and the results are set out in Table VIII. The agreement with the standardised mortality ratio is again reasonably good, especially in regard to female deaths.

TABLE VIII.—*Relative Intestinal Cancer Mortalities*

| | Male | Female |
|---|---|---|
| Expected deaths (based on England and Wales rates) | 15·39 | 16·5 |
| Expected deaths due to older age of the population in Llanfyllin R.D. | 1·54 | 1·4 |
| Total expected deaths | 16·93 | 17·9 |
| Actual deaths | 26·0 | 26·0 |
| Actual deaths as percentage of expected deaths | 153·6 | 145·3 |
| Standardised mortality ratio | 142·9 | 149·8 |

Since the period 1931–39 there has been a steady decrease in intestinal cancer mortality in England and Wales until in 1950–54 it was 78·2 per cent (male) and 76·9 per cent (female) of its former level. The standardised rates for England and Wales had been building up to a peak in the years 1931–39, but even these rates were less than those prevailing in Llanfyllin R.D. It cannot be argued therefore that the high Llanfyllin rate is merely a local failure in the national mortality decline. It is more likely to have been above national levels at all times and to have been thrown perhaps into greater relief recently with the declining mortality elsewhere.

The distribution of the sex and age specific death rates are shown in Table IX in which the heavier local death rate is seen to be reflected in nearly all the main age groups. The average age at death is a little higher in intestinal than in stomach cancer (Table X)

TABLE IX.—*Intestinal Cancer: Sex and Age Specific Death Rates per Million in Llanfyllin R.D. During* 1946–59 *and in England and Wales During* 1946–57

| | Age groups | | | | | |
|---|---|---|---|---|---|---|
| | 35–44 | 45–54 | 55–64 | 65–74 | 75–84 | 85 and over |
| Male : Llanfyllin R.D. | 99 | 199 | 797 | 1610 | 2566 | 2331 |
| England and Wales | 44 | 130 | 392 | 1127 | 2273 | 2592 |
| Female : Llanfyllin R.D. | — | 202 | 377 | 1545 | 4596 | 2008 |
| England and Wales | 52 | 162 | 409 | 952 | 2064 | 2963 |
| Actual numbers of deaths : Male | 1 | 2 | 6 | 10 | 6 | 1 |
| Female | — | 2 | 3 | 9 | 11 | 1 |

TABLE X.—*Average Age at Death*

| | England and Wales | | Llanfyllin R.D. | |
|---|---|---|---|---|
| | Male | Female | Male | Female |
| | (years) | (years) | (years) | (years) |
| Stomach cancer | 66·16 | 69·8 | 66·6 | 69·1 |
| Intestinal cancer | 68·8 | 70·0 | 67·1 | 72·1 |

*Note :* The national figures are based on statistics for the period 1950–55 obtained from Studies on Medical and Population Subjects. No. 13 (General Register Office), using the mid-1953 population, and the local figures apply to the years 1946–59.

The female 75–84 age group, which had the highest number of deaths has no obvious common factor. The deaths are distributed evenly throughout the period of time, geographically they are evenly distributed, and their proportions in the social classes are similar to those of the deaths as a whole.

As in the case of stomach cancer, Social Classes II and IV consist mainly of farmers and agricultural workers and Table XI shows again a tendency to higher standardised mortality ratios for these occupations, especially farmers. It also shows that intestinal cancer, unlike stomach cancer, has a tendency to affect Classes I and II more than IV and V. The same applies to Llanfyllin R.D. where there were nearly as many deaths due to intestinal as to stomach cancer in Classes I and II. Certainly, taking these two classes together, there is a greater proportion of intestinal than stomach cancer (Table V and Fig. 2).

The over-all percentage of cancer of the intestine in the male agricultural population is appreciably greater than the percentage that that population bears to the total occupied males (Table VII). The same explanation for the disparity between the rates for farmers and agricultural workers in regard to stomach cancer applies to this cause of death in that many agricultural labourers also have a few acres of ground and so may be classified as small farmers. The female rate attributable to farming occupations is not so convincing however and yet, knowing

TABLE XI.—*Intestinal Cancer: Standardised Mortality of the Social Classes in England and Wales*, 1951

| | Social class | | | | |
|---|---|---|---|---|---|
| | I | II | III | IV | V |
| Males aged 20–64 years | 123 | 100 | 105 | 85 | 94 |
| Farmers | — | 117 | — | — | — |
| Agricultural workers | — | — | — | 90 | — |
| Females aged 20–64 years | 103 | 100 | 97 | 104 | 106 |
| Farmers | — | 113 | — | — | — |
| Agricultural workers | — | — | — | 100 | — |
| Intestinal cancer death rates per 10,000 all causes | | | | | |
| Males aged 65 years and over | 210 | 211 | 192 | 187 | 192 |
| Farmers | — | 214 | — | — | — |
| Agricultural workers | — | — | — | 198 | — |
| Females aged 65 years and over | 375 | 291 | 262 | 271 | 275 |
| Farmers | — | 254 | — | — | — |
| Agricultural workers | — | — | — | 277 | — |

the excessive mortality among females, it may well be understated. There were six among those classified under other occupations who could have had farming associations stronger than the mere fact of living in a farming community. This also may apply to stomach cancer deaths only not to the same extent.

*The other districts*

The study was extended to the other four districts of North Montgomeryshire covering the period 1953–59 for the Borough of Welshpool and Forden R.D. and 1954–59 for the Boroughs of Montgomery and Llanfyllin. Three of these districts contained small concentrations of old people sufficient to affect the death rates of each district. Taken together however, there was no appreciable effect of this kind because most of these old people originated within the five districts forming the area of this enquiry. The Borough of Llanfyllin has accommodation for old people (59 at the time of the census), and this is reflected in the high proportion of the population over the age of 65 years (Table IV). These 59 people represented about 0·5 per cent of the combined population of the Borough and Rural District of Llanfyllin, yet they contributed to cancer deaths and stomach cancer deaths to the extent of 3 and 4 per cent respectively. On the other hand Welshpool, though possessing a small home for old people (about 12), was the only district with a proportion of persons over 65 years similar to that in England and Wales. The social class representation is also similar to that in England and Wales (Fig. 1 and 4), both for population and cancer mortality. A hospital for mental defectives in the Forden R.D. (140 inmates at the time of the census) also has a department for the chronic sick of Forden and the other four districts. Less than twenty beds were being used for this purpose during part of the time covered by this study.

The comparative rates for gastro-intestinal cancer in these three districts are shown in Table XII. When compared with the rates for Llanfyllin R.D. shown in Table I, it will be seen that, despite the concentration of old people in the other districts, Llanfyllin R.D. has virtually the highest rates. The more strictly comparable mortalities for cancer of the stomach in Llanfyllin R.D. during the period 1953–59 were 581 (male) and 378 (female) deaths per million.

The representation of social class is given in Table XIII and Fig. 3 and 4. As in Llanfyllin R.D., the farming community is relatively more subject to cancer of the gastro-intestinal tract than to cancer of other sites. Farmers again show a

**Fig. 1.**—Proportionate representation of population and stomach cancer among social classes. *Note :* The population relates to the year 1951 and cancer to the years 1946–57. England and Wales.

**Fig. 2.**—Proportionate representation of population and cancer among social classes. *Note :* The population relates to the year 1951 and cancer to the years 1946–59. Rural District of Llanfyllin.

high rate for stomach cancer but, when grouped with farm workers, their contribution to the total deaths from this cause is not much in excess of the joint proportion of these two occupational groups in the population.

Population and cancer distribution in the rural districts are similar both for the 1946–59 period in Llanfyllin R.D. and the 1953–59 period in Forden R.D. On the other hand Welshpool shows a distribution remarkably like that of England and Wales (Fig. 1 and 4). Welshpool itself is about fifty per cent rural however, and when the total cancer deaths in the 1953–59 period are grouped strictly according to urban or rural residence, it is found that, while their general distribution is equal, there is a preponderance of deaths from stomach cancer among those with truly rural places of residence.

Cancer deaths in Welshpool, 1953–59

| | Urban residence | Rural residence |
|---|---|---|
| All sites . . . . . . | 38 | 39 |
| Other cancer of digestive system . | 14 | 12 |
| Stomach cancer . . . . . | 1 | 6 |

FIG. 3.—Proportionate representation of population and cancer among social classes. *Note :* The population relates to the year 1951 and cancer to the years 1953–59. Rural District of Forden.

Fig. 4.—Proportionate representation of population and cancer among social classes. *Note :* The population relates to the year 1951 and cancer to the years 1953–59. Borough of Welshpool.

TABLE XII.—*Gastro-intestinal Cancer Mortality : Deaths per Million in the Districts of North Montgomeryshire in* 1953–59, (1954–59 *for the Boroughs of Montgomery and Llanfyllin*)

| Site | Welshpool M. | Welshpool F. | Forden M. | Forden F. | Llanfyllin Borough M. | Llanfyllin Borough F. | All areas except Llanfyllin R.D. M. | All areas except Llanfyllin R.D. F. |
|---|---|---|---|---|---|---|---|---|
| Oesophagus | — | 46 | 288 | — | — | 237 | 137 | 45 |
| Stomach | 302 | 46 | 577 | 473 | 736 | 1425 | 434 | 354 |
| Intestine | 151 | 329 | 233 | 297 | 491 | — | 228 | 284 |
| Rectum | 151 | 46 | 346 | 178 | — | 237 | 206 | 133 |
| Digestive system | 1008 | 560 | 1615 | 1008 | 1963 | 2136 | 1347 | 927 |
| All sites | 2164 | 1590 | 2537 | 1722 | 2453 | 4751 | 2353 | 1990 |

TABLE XIII.—*Population and Cancer Distribution Among the Social Classes of the Four Areas (the Boroughs of Welshpool, Llanfyllin and Montgomery and Forden R.D.) : Population per* 1000 *occupied and Retired Males,* 1951, *and Corresponding Distribution for Cancer in* 1953–59 (1954–59 *for the Boroughs of Montgomery and Llanfyllin*)

| | Social class I | II | III | IV | V |
|---|---|---|---|---|---|
| Population | 28 | 310 | 341 | 215 | 106 |
| Cancer all sites | 47 (9) | 358 (69) | 306 (59) | 140 (27) | 149 (29) |
| Farmers and farm workers | — | 229 (44) | — | 86 (17) | — |
| Cancer of stomach | 29 (1) | 429 (15) | 257 (9) | 86 (3) | 199 (7) |
| Farmers and farm workers | — | 371 (13) | — | 86 (3) | — |
| Cancer of intestine | 43 (1) | 478 (11) | 348 (8) | 88 (2) | 43 (1) |
| Farmers and farm workers | — | 320 (8) | — | 44 (1) | — |

*Note :* The numbers in brackets refer to actual deaths.

Owing to the low numbers, undue significance cannot be attached to this finding, but it lends support to the association of stomach cancer with the more rural areas and perhaps with some widely-spread environmental factor. Possibilities of this nature will be discussed later. Histograms have not been included for the Boroughs of Llanfyllin and Montgomery owing to their small numbers. The addition of the Llanfyllin Borough mortality to that of the Rural District of Llanfyllin does not affect the latter materially, and a combination of the other three district mortalities is merely a compromise of the mortalities of Welshpool and Forden R.D.

### *Genetic considerations*

In patients with stomach cancer blood group A was found to be commoner than in the general population (Aird *et al.*, 1953), and this excess of group A was thought to be associated only with carcinomata of the pyloric end of the stomach (Jennings, Balme and Richardson, 1956), and with blood group A1 rather than A2 (Walther, Raeburn and Case, 1956). The question arises as to whether or not an unusual distribution of these groups exists in Mid-Wales. In her paper given to the British Association in 1956 Dr. Ada C. Kopeć gave the A, B and O gene frequencies in thirteen regions of Great Britain including that of North Wales, though not actually including Montgomeryshire. The gene A frequencies varied from 19·87 per cent in Western Scotland to 29·44 per cent in Sussex with North Wales occupying an intermediate position at 24·79 per cent. In another survey the gene O frequencies were found to rise on proceeding northwards and for similar latitudes they were higher in Wales than in Western England (Watkin, 1956). In the latter survey sub-group A2 was found to be unusually prevalent in Mid-Wales, being 35 per cent and nearly 50 per cent of group A in two population samples in North and Mid-Wales, compared with 22 per cent in Southern England. The geographical distribution of blood groups in the ABO system does not appear to influence in any way the incidence of stomach cancer in North Wales.

*Geochemical considerations*

Some of the more radioactive rocks in the British sedimentary sequence are to be found in North Wales. The highest uranium contents are to be found in certain black shales in rocks which include those of Upper Cambrian age (Ponsford, 1955). In the main the radioactivity is due to the content of uranium and it is customary to express the total radioactivity as uranium oxide equivalent. It has been found that the average shale is about four to six times as active as the average limestone. These black shales have been proved to be radioactive at all known localities but the activity and uranium enrichment are at the most only one quarter of those reported for the equivalent Swedish rocks. These rocks are to be found most prominently to the north and west of the Bala fault. To the south and east, which includes Montgomeryshire, the geological formation is largely of Silurian age and musuem specimens of black shales and phosphates of Silurian and Ordovician age have been found to possess a low radioactivity, though up to twice that of an average shale. The well-known phosphate deposit of comparable age at Pen-y-Garnedd in the Rural District of Llanfyllin is not radioactive (Wedd, *et al.* 1929). Great thicknesses of these black shales have not been adequately sampled however, and there is a possibility that other radioactive beds may be present.

The most obvious manner in which the radio-activity of these rocks could be further concentrated and communicated to populations would be through the drinking water. The distribution of uranium in a drainage system has been studied in the Fal River in Cornwall where it was found that the uranium oxide content in grams $\times 10^{-6}$ per litre rose from levels between 0·7 and 7·5 to levels between 1·0 and 25·0 after a prolonged rainy period which had been preceeded by several months of comparatively low rainfall (Ostle, 1954). The variation was attributed to the action of acidic water upon uranium followed by the leaching effect of subsequent sustained rainfall. As similar conditions were thought to apply in Mid-Wales, it was decided to investigate the water supplies of some of the places of residence where deaths from cancer of the stomach had occurred. The chief public water supplies of North Montgomeryshire were dealt with in like fashion. Using the quantitative field method for the determination of uranium in natural waters described by Ostle (1954), results were obtained which are given in Table XIV.

It is interesting to note that the average of the results shown in Table XIV is equivalent to 0·2 $\mu\mu$c, per litre (range 0·03 to 0·6 $\mu\mu$c per liter) as compared with 0·45 $\mu\mu$c per litre, 0·24 $\mu\mu$c per litre and 0·01 $\mu\mu$c per litre strontium-90 in lake, river and well water supplies respectively in 1957–58 (Stewart *et al.*, 1959). As

TABLE XIV.—*Uranium Oxide Content of Certain Water Supplies in North Montgomeryshire*

| Code number | Locality | Type of source | Uranium oxide content |
|---|---|---|---|
| | | Public supplies | |
| 1 | Llanerfyl | Springs | $0 \cdot 6 \times 10^{-6}$ g. per l. |
| 2 | Llanfair Caereinion | Banwy River | 1·4 ,, ,, |
| 3 | Meifod | Springs | 0·5 ,, ,, |
| 4 | Dolanog | ,, | 0·6 ,, ,, |
| 5 | Llangynog | ,, | 2·0 ,, ,, |
| 6 | Llanfyllin | ,, | 0·6 ,, ,, |
| 7 | Llanfechain | ,, | 1·1 ,, ,, |
| 8 | Llansantffraid | ,, | 0·8 ,, ,, |
| 9 | Welshpool | | |
| | (i) Trinity Well | ,, | 0·9 ,, ,, |
| | (ii) Black Pools | Springs and surface water | 0·6 ,, ,, |
| 10 | Foel | Springs | 0·2 ,, ,, |
| 11 | Penybontfawr | ,, | 0·1 ,, ,, |
| | Private residences where a death from cancer of the stomach had occurred | | |
| 12 | Pen-y-Garnedd | Shallow well | $0 \cdot 6 \times 10^{-6}$ g. per l. |
| 13 | Hirnant | Springs | 0·8 ,, ,, |
| 14 | Llanwddyn | Lake Vyrnwy | 0·6 ,, ,, |
| 15 | Llanfihangel | Shallow well | 0·9 ,, ,, |
| 16 | Bwlchycibau | ,, ,, | 0·6 ,, ,, |
| 17 | Llanymynech | ,, ,, | 0·9 ,, ,, |
| 18 | Guilsfield | ,, ,, | 0·8 ,, ,, |
| 19 | Pentrebeirdd | Springs | 0·9 ,, ,, |
| 20 | Llanfair Caereinion | Shallow well | 0·9 ,, ,, |
| 21 | Penybontfawr | ,, ,, | 0·2 ,, ,, |
| 22 | Pontrobert | ,, ,, | 0·2 ,, ,, |
| 23 | Llandrinio | ,, ,, | 0·7 ,, ,, |
| 24 | Llanfair Caereinion | ,, ,, | 0·1 ,, ,, |
| 25 | Sarnau | ,, ,, | 0·2 ,, ,, |

*Note :* Sample numbers 10, 11, 18 and 21–25 were subject to repeat analysis by two different techniques and by two different analysts. Agreement was good considering the low content of uranium and in each case the greater of the two results is quoted.

far as the uranium content of the samples is concerned, serious significance can hardly be attached to such extremely small amounts of radiation especially as the uranium contents are no higher than would be expected in most natural waters. The highest bone activity due to strontium-90 in children under five years in 1956 was only one tenth of that due to natural radium when allowance had been made for the relative biological efficiency of the alpha rays, and it was about one thirtieth of the total natural dose to bone from internal and external sources (Bryant *et al.*, 1958*b*).

These results do not take into account the daughter elements however, or the great quantitative variation that may develop among them. Two examples of this variation, yet of different types, have been described recently in Devon and Wales. High activities of 0·1 $\mu\mu$c per ml. to 13·3 $\mu\mu$c per ml. have been recorded for waters in Devon (Abbatt *et al.*, 1960). These are presumably total radiations including beta as well as alpha activities and the thorium series as well as the uranium series ; nevertheless gamma spectrometry identified radon as being at least primarily responsible for the gamma activity of the samples. The ratio of thorium-232 to uranium-238 in a sample of Devon soil was 1·3 and the total soil activity was about three times that of the samples from Wales (Mayneord, Turner and Radley, 1960). In Wales an aliquot of raw matt which showed increasing uranium series activity was found to have a lead-210 content twenty-seven times higher than the radium-226 level (Mayneord *et al.*, 1960). The difference between Wales and Devon as far as alpha activity is concerned is that, although in both Devon and Wales the soil showed small rises of 5 to 6 per cent in activity on sealing for about six months, in Wales and in Scotland the grass ash activities after six months' storage showed much higher values than in Devon due to the growth of polonium-210 (Mayneord *et al.*, 1960).

Two mechanisms are thought to be at work. The first is the leaching effect of underground waters of differing pH values upon uranium and thorium ores of varying qualities and of varying degrees of accessibility. Such waters, dependent also upon drainage and depth below the surface, may leach out certain members of the series more than uranium itself. In particular the radons-222 and -220 imprisoned in varying concentrations may be released in varying amounts. If small amounts of radium D (lead-210) were to be selectively released, the ratio of the disintegration constant of its main precursor radium-226 and its own constant (1 : 135) would ensure a similarly enhanced degree of radioactivity. The second mechanism is the retention of lead-210 from the atmosphere in proportion to rainfall after the fashion of the foliar retention of strontium-90. The basic uranium and thorium activities of soil samples in Wales were less than in Devon and certainly the uranium content of the water samples was lower so that it is possible that the second mechanism is of greater importance in Wales than in Devon. If a causative association is to be entertained between radioactivities and gastric cancer, then both of these mechanisms are likely to be relevant to the problem although, for reasons to be discussed, the second may be the more significant in Wales.

In Sweden, where radioactive shales are several times more active than in Wales, there is evidence of a gastric cancer mortality which is not only higher than the average but also constitutes a much great erproportion of cancer of the digestive system than in England and Wales. It must be admitted however that the high specific death rates for cancer of the stomach at ages over 75 years contributed substantially to the high total in Sweden (Table XV).

TABLE XV.—*Standardised Cancer Death Rates per Million of Population for Cancer of the Digestive System and Stomach only in Sweden and England and Wales*

| | Male | | Female | |
|---|---|---|---|---|
| | Cancer of digestive system and peritoneum | Cancer of stomach | Cancer of digestive system and peritoneum | Cancer of stomach |
| Sweden, 1951–55 | — | 433 | — | 322 |
| 1951–53 | 840 | — | 707 | — |
| England and Wales, 1946–57 | — | 382 | — | 275 |
| 1951–53 | 953 | — | 800 | — |

Quantitative tests were carried out on the public water supplies in order to estimate the fluoride content and in each case the amount was negligible or of the order of 0·1 parts per million.

### DISCUSSION

The mortality from cancer of the stomach in England and Wales has been declining over the period 1936–54 but not in the age-groups over 75 years. On the other hand the comparative mortality index for Wales rose by 5·4 per cent (male) and 5·6 per cent (female) between the quinquennia 1946–50 and 1951–55. Again, Wales differed from England and Wales in having a higher standardised mortality ratio for stomach cancer in rural than in urban areas (General Register Office, 1957). This feature of the mortality can be seen in miniature in the Welshpool figures of the present study. The latest standardised mortality ratios for Wales (for 1958) are 130 (male) and 159 (female) compared with 93 and 107 respectively for cancer of all sites (Registrar General, 1958).

The pattern of gastric cancer appears to be related to geographical regions such as Wales and the Northern region of England. The high rates prevailing in these areas must have contributed appreciably to the fact that England and Wales had the fifth highest mortality from cancer of the digestive organs and peritoneum during the years 1951–53 in a list of twentyone countries. (The death rates were made available by the Institute of Cancer Research.) Germany, Switzerland, Scotland and France occupied the first four consecutive places on this list followed by England and Wales, Denmark, Finland, Norway, Sweden and Ireland. It must be observed that all these countries except Finland had older populations than the remaining eleven countries in which the populations over the age of 50 years amounted to less than 18 per cent. Finland, Japan and Chile had fairly high specific rates for the ages 50–65 however, and apart from this broad division of the countries into two groups, there was no detailed correlation between the numbers of old people and the mortalities. Local investigations within countries, whether statistical or otherwise, are more likely to reveal possible causal relationships than the rather unwieldy national statistics. Cancer of the stomach for females in Wales for the years 1947–53 has been accorded such treatment by the geographer, and his map of Wales shows that nearly all of the rural counties including Montgomeryshire have standardised mortality ratios ranging from one and three-quarters to twice the national average ; in fact the map focuses attention upon rural districts *per se* because so many of the small urban districts within the large rural districts, including those in Montgomeryshire, have lesser mortalities (Howe, 1959). The annual report of the Medical Officer of Health for Carmarthen (Evans, 1959) contains statistics which show that the gastric cancer death rate for that county was 486 per million or 23·6 per cent of the total cancer death rate. In 1958, stomach cancer in England and Wales amounted to 15·6 per cent (male) and 13·7 per cent (female) of the total cancer deaths (Registrar-General, 1958).

Investigations have now been carried out on an even more local basis by means of the analysis of soil samples from the gardens of houses where a case of stomach cancer was known to have occurred, comparing the results thus obtained with those of control samples. In Caernarvonshire, Merionethshire and Cheshire, garden soils taken from houses where a person has died of stomach cancer after fifteen years or more of residence, have been found to have higher median concentrations of zinc and chromium than garden soils from houses in the same counties where a person has died from a cause other than cancer or has died of cancer after residence of less than two years. In Welsh districts with high stomach mortality, but not elsewhere, the incidence of gastric cancer after 10–19 years of residence was also found to be excessive where the garden soil had a high organic carbon content and even more so after 20 years or more of residence where the soil had a medium organic carbon content (Stocks, 1958). Zinc is one of the trace elements shown to be essential for the higher forms of animal life, but chromium is not even among the apparently unessential elements known to be normally present in the body tissues (Underwood, 1956). It was therefore all the more significant that a local high gastric cancer rate in a township in Devon, situated in a district which had an average gastric cancer rate, was found to be associated with a high chromium content of the garden soils of the houses in which a case of stomach cancer had occurred. Furthermore the excess of chromium, zinc, cobalt, nickel and organic matter at the stomach cancer addresses exceeded the respective control levels to a significant degree in two sub-districts in which there were tanneries (Stocks and Davies, 1960*a*). Both tanneries used chrome and the deaths occurred among those who might have been expected to eat much of their own garden produce (Nicholson, 1960). A positive association has also been found between chromium content of the soil and intestinal cancer (Stocks and Davies, 1960*b*), but in the Rural District of Llanfyllin no relationship between intestinal cancer and the occupation of tanner or the existence of tanneries was found. Samples of soil of course might have revealed high chromium contents. The importance which might be attached to micronutrient deficiencies or to carcinogenic contaminants of vegetable foods in North Wales (Stocks, 1959 ; Davies and Griffith, 1954 ; Legon, 1952) has been doubted on the grounds that, with the exception of potatoes, 78–85 per cent of vegetables are brought into North Wales (Howe, 1959).

Whether this mode of direct conveyance of carcinogens to the body by vegetable foods is important or not, many workers have concentrated attention upon the other and main component of the diet, namely water. London boroughs supplied by well-water had lower cancer mortalities than most of the boroughs supplied by river water (Stocks, 1947). Similar findings in the Netherlands stimulated work on soil analysis there (Tromp and Diehl, 1955), yielding results not unlike those in North Wales (Davies and Griffith, 1954 ; Stocks, 1958). In both cases the higher gastric mortalities were associated with acid poorly drained peaty soils of the type likely to produce waters having a solvent action in piped supplies. Treated and untreated water supplies in rural Wales are generally soft or moderately so and, with several exceptions, the softest waters occur in the highest gastric cancer-bearing areas (Howe, 1959). In Montgomeryshire the southern half of the county has fairly hard water supplies although the raised gastric cancer mortality applies generally to the rural districts throughout the county. The water supplies in the Llanfyllin R.D. are mostly soft apart from two treated supplies whose treatment includes passage through a contact chamber of limestone chippings. Despite a gradual improvement since 1946 in the provision of public mains in this district, the section of the population served in this way in 1957 was still only 38·7 per cent. Many of the public supplies are virtually untreated waters which nevertheless reach the necessary bacteriological standards for drinking water. An attempt was made to ascertain differences in gastric cancer mortality related to public or private water supplies but none were found. It should be noted however that Liverpool's water supply is derived from Lake Vyrnwy whose catchment area extends over much of the rural district of Llanfyllin, but in this case an excess of stomach cancer in Liverpool appears only amongst females (S.M.R. 128 in 1950–54), and of course the water is subjected to the full treatment process not far from the city. A negligible amount of water from Lake Vyrnwy is used in Llanfyllin R.D. itself. As an indication of the potential contamination of private supplies in this district 20 out of 29 samples were found to be either unsatisfactory or suspicious on bacteriological examination in 1957 compared with 10 out of 205 samples from treated public supplies and 59 out of 318 samples from untreated public supplies. These private supplies were usually tested after protective works had been carried out in preparation for a grant-aided farm water scheme or the erection of a new house, so that the results may have indicated better conditions than actually prevailed. Recent experimental work justifies alertness to the possibilities of water contamination. Because it was thought possible that even weakly acting carcinogenic agents ingested over a long period might be dangerous, mice were fed 25 $\mu$g. 3,4-benzopyrene for 400 days with or without surface-acting agents. Definite carcinoma of the stomach was produced only in the group of mice which had been fed with a normal diet but had received benzopyrene in their drinking water to which detergent had also been added (Borneff, 1960).

Mutagens, which have much in common with the chemical carcinogens, also merit some consideration. Whilst the fall-out of strontium-90 cannot be expected to have a significant bearing, if any at all, upon the present study, the numerous papers on strontium-90 provide valuable information much of which can be applied to the allied subject of natural fall-out. In the breakdown of uranium the emanation of radium (Radon-222) escapes from rock and soil in varying degrees throughout the world. The rate of escape of this element is highest in the dry equatorial regions from which it tends to rise with upward air currents towards the stratosphere. The rapid change through four radioelements is over 90 per cent complete in about two weeks with the formation of lead-210 which has a half-life of nearly 19·5 years. Being particulate, this element reaches the ground as fall-out in temperate regions after approximately four weeks in the atmosphere, a period sufficient for appreciable atmospheric mixing to occur, yet short compared with its radioactive half-life (Burton and Stewart, 1960). The model of global circulation of air (Dobson, 1956) and the world-wide measurement of long-lived fission products (Stewart *et al.*, 1957) help to explain the general pattern of deposition which, although more consistently related to the concentration of these products in air or rain, is in the final distribution roughly proportional to rainfall (Peirson, Crooks and Fisher, 1960). The areas of rainfall in the United Kingdom can be divided as follows :—

| | |
|---|---|
| Over 60 inches : | West Highlands |
| | Much of Wales |
| | Much of Cumberland |
| | Three small areas in Devon |
| 30–60 inches : | Western half of Great Britain |
| | Northern Ireland |
| Under 30 inches : | Eastern half of Great Britain |

The high level of basic top-soil activity in Wales, coupled with the restricting influence of an even moderate rainfall upon the rate of escape of radon-222 from the surface of the soil (Jaki and Hess, 1958) accentuate the effects of rainfall itself. The lead-210, whose content in one matt specimen was found to be twenty-seven times higher than the radium-226 level, eventually becomes polonium-210 with a half-life of 138·7 days and a radiation of the alpha type. Polonium-210 forms the chief natural activity in pasture plants, in fact about 84 per cent (Mayneord *et al.*, 1960).

It is relevant to compare strontium-90 and lead-210 deposition. In 1956–57 the deposition of strontium-90 was 2600 $\mu\mu c$ per square metre at Milford Haven. The deposition of lead-210 was calculated to be 2260 $\mu\mu c./m^2$ per per year per 100 cm. rainfall, which might mean anything from 3000 to 4000 $\mu\mu c./m$ in the upland areas of Wales (Burton and Stewart, 1960). Strontium-90 is particularly well entrapped in the slow-growing matted pastures characteristic of the relatively unproductive hill areas. The soil is uncultivated and peaty and there is a low yield of vegetation and the strontium-90 tends to remain substantially in the topsoil for years so that the uptake of these elements by animals is bound to be high. In 1956 the measurements per square metre of the surface and per gramme calcium in soil and grass were all many times higher in these upland areas than elsewhere. It was not surprising therefore to find readings of 183 and 35 $\mu\mu c$. per g. calcium in sheep bone at Cwmystwyth and Welshpool respectively, in contrast to readings of 7 to 15 $\mu\mu c$. per g. calcium for lowland sheep (Bryant *et al.*, 1958*a*, 1958*b*). Total soil alpha activities were found to be 15 times higher in Wales than in South-East England and the total grass ash alpha activities were also many times higher (Mayneord *et al.*, 1960). As the proportion of strontium-90 per gramme calcium is about 100–300 times higher in grass grown on calcareous soil than in the soil itself, the dominant mode of contamination is by means of foliar retention (Bryant *et al.*, 1958*a*, 1958*b*). This is also borne out by the ratios of strontium-90 to stable strontium and calcium in flour, which were consistently lower than those for bran which is more accessible to current fall-out (Agricultural Research Council, 1958). The same also applies to polonium-210 because the total alpha activities for the outer parts of the plant such as the leaf and bark have been found to be much higher than those for the fruit and root (Mayneord *et al.*, 1960). Despite the fact that the thorium series contributes more than the uranium series to soil activity—and in Wales soil samples were found to have Th/Ur ratios of about 4·5—the plant has a preference for the radium isotopes which have been found to contribute up to 90 per cent of the total alpha activity in the plant (Mayneord *et al.*, 1960). Just as a biological discrimination against strontium-90 may amount to a factor of 25 to 30 from vegetation to the bone of a child (Bryant *et al.*, 1958*a*), there is also likely to be discrimination against polonium. The difference between readings in samples of dry grass (2·6 to 26 $\mu\mu$ c. per gramme dry grass) and in samples of lamb kidney (of which the highest reading was 2·38 $\mu\mu c$. per gramme wet cortex tissue) in two Welsh towns, gives a factor of anything between 1 and 11 (Hill, 1960). Such low discrimination is of course balanced by the shorter half-life and lower body retention of polonium.

The level of activity on entry to the food chains is likely to be much higher in Wales due to the greater rainfall and cumulative effects. Because foliar retention is so much greater it was not surprising to find that only a short period of time elapsed between the deposition of strontium-90 and its appearance in milk. Measurements of strontium-89 in relation to strontium-90 in milk and rainwater confirmed that this period was only about one or two months. In 1956 the amount of strontium-90 in milk (4·4 $\mu\mu c$. per litre and also per gramme calcium) was nearly twice that found in rainwater (Bryant *et al.*, 1958*b* ; Bryant, Morgan and Spicer, 1958). The ratios of strontium-90 to calcium in blood and milk of animals were found to be respectively 0·25 and 0·12 of the ratio in the diet. Slaughtered animals which have been raised on dairy pastures will have twice the ratio of strontium-90 to calcium as that found in milk. At stations in Cardiganshire and Merionethshire in 1959 however, the proportions of strontium-90 to calcium in the milk were 33 and 49·9 $\mu\mu c.$ per gramme calcium respectively. Where beef cattle, grazed on acid unimproved upland soils, are concerned, and even more so in relation to sheep, high strontium-90 readings are to be expected in the beef and mutton. In 1959 meat only accounted for about 3·5 per cent of the strontium-90 in the human diet in the United Kingdom (Agricultural Research Council, 1959), but a tenfold increase in this proportion, as might occur in Welsh meat, could increase the total intake by a quarter. In Wales as elsewhere milk is mainly derived from dairy farms situated in the more fertile lowland areas in Wales and Western England. Meat on the other hand is more likely to be derived from hill cattle and sheep. In Llanfyllin R.D. local slaughtering is common although in border areas meat also tends to be bought " off the hook " from centres such as Shrewsbury. Polonium-210 may therefore gain access to the body disproportionately through meat in Welsh districts.

The alpha activities of certain foodstuffs have been ascertained (Mayneord, 1959). In some cases the figures obtained can be compared directly with the beta activities of strontium-90 in 1959, but no account has been taken either of the tenfold relative biological efficiency of the alpha radiation or the concentration of strontium-90 in bone (Table XVI). It has been estimated that over 96 per cent of

TABLE XVI.—*Radioactivities of Certain Foodstuffs*

| Foodstuff | Maximum alpha activity ($\mu\mu c.$ per 100 g.) | Activity due to strontium-90 (year 1959) ($\mu\mu c.$ per 100 g.) |
|---|---|---|
| Cereals | 60 | — |
| Cereal-based infant foods | — | 4·1 |
| Teas | 40 | 39·7 |
| Flours | 14 | 1·56 |
| Wholemeal flour | — | 3·8 |
| Milks (evaporated) | 1 to 2 | 2·3 |
| Eggs | 0·9 | 0·52 |
| Vegetables | 0·7 | 1·0 |

the total strontium-90 intake is dietary (Agricultural Research Council, 1959). The daily alpha activities of diets, though of a small order, may vary, according to the foods chosen, by a factor as high as a thousand (Mayneord, 1959). Having gained access to the body it would have to be supposed that polonium-210, and for that matter any carcinogenic agent, would undergo some form of concentration in the stomach, perhaps in the cancer-prone pyloric region. Studies of the distribution of polonium in animals and in the human body did not reveal any abnormal concentration in the stomach, but the amounts of polonium being administered were almost of pharmacological proportions (200–300 $\mu\mu c.$ per g. body weight) compared with the amounts being considered here. There were high concentrations in the liver and kidney however, the latter being associated with clearances that were considerably lower than for some similar metals (Fink, 1950).

Two characteristics of polonium compounds are that they are not ionised and that they can separate in the colloidal state (Sidgwick, 1950). Such compounds might be expected to show resemblances to the other members of their chemical sub-group which includes sulphur and selenium. Most of the selenium in wheat is associated with the protein and is therefore well distributed throughout the grain especially in the bran. In organic form in cereal grain it is highly toxic and it is thought that it may react with the sulphur-containing amino acids, partially replacing the sulphur. In animal tissues it concentrates in liver, muscle and the gastro-intestinal tract (Underwood, 1956). Just as cobalt forms four per cent of the molecule of vitamin $B_{12}$, so polonium might enter the body in similar fashion or, like selenium, it might supplant other elements in the enzyme systems, especially the cytochrome system which may govern the production of hydrochloric acid in the stomach. The radioactivity of polonium-210 might be expected to enhance or exceed the effect of any toxicity inherent in the element itself.

Cellular injury initiates growth and repeated injury may profoundly alter the processes both of agamic reproduction and of regeneration. Hyperplasia blends imperceptibly into neoplasia like graded manifestations of tissue malformations (Smithers, 1959). Carcinogenesis also appears to have an initiating phase and a promoting phase with most of the chemical carcinogens combining both phases. The cellular change induced by an initiating substance is irreversible, instantaneous and invisible (Berenblum and Shubik, 1949). A somatic mutation, such as might be caused by an alpha radiation is likely to produce the first of these phases and bring on the second phase more rapidly than other forms of cellular injury. It is certain that only microgramme quantities of the more active carcinogenic compounds are required to produce tumours in mice and human cancer also may be produced either by small amounts of highly active compounds, perhaps acting for a relatively short time, or larger amounts of weaker agents acting over many years (Cook, 1957).

The finding of an increased mortality for cancer of the intestine in the Llanfyllin R.D. is contrary to expectations because in 1950–54 the standardised mortality ratios for cancer of the intestine and rectum in the rural districts of Wales were only 96 (male) and 105 (female) (General Register Office, 1957), and in 1950–52 in the three counties of Montgomeryshire, Cardiganshire and Breconshire they were 90 (male) and 94 (female) (Griffith, personal communication). In view of the statistical evidence against an increased intestinal cancer death rate in Wales, the figures given for Llanfyllin R.D. cannot attract other than sceptical comment owing to the small numbers involved ; nevertheless the excess was of similar proportions to that for the stomach over the same period. The extension to the intestine of the same carcinogenic influence that makes stomach cancer mortality excessive in Wales is possible ; indeed it is perhaps surprising that in Wales the whole of the digestive system is not equally affected.

The validity of the diagnoses on the death certificates in North Wales is such that there has been no over-statement of stomach cancer mortality and the incidence of the disease is no higher amongst people of Welsh parentage than amongst those of English parentage living in the same districts (Stocks, 1958). In Llanfyllin R.D. itself no evidence was found that certification had been inaccurate either recently or twenty to thirty years ago.

### SUMMARY

The study of gastric cancer mortality in North Montgomeryshire commenced with the Llanfyllin R.D., being the largest of the five constituent authorities, but eventually it was extended so as to cover all five authorities. The total cancer rate for the Llanfyllin Rural District during the period 1946–59 only slightly exceeded that for England and Wales during an almost similar period. The excess would have been greater but for the high rates for lung cancer in males and cancer of the genital system in females in England and Wales. When the cancer sites were examined individually however, it became obvious that this district, in common with the rest of rural Wales, possessed a high mortality rate for gastro-intestinal cancer, high enough in fact to off-set the national rates for male lung cancer and female genital cancer. The standardised mortality ratios in Llanfyllin R.D. were found to be 135 (male) and 161·5 (female) during the period 1946–59. The apparent disparity between the sexes was due largely to the excess of older men in the community, thus strengthening the view that a general carcinogenic agent might be at work.

There was a suggestion of earlier death from this cause but no specific age group or occupation was involved in the increase and the social variations tended to strike a balance between stomach and intestinal cancer so that the total for both was evenly distributed over all the social classes. Farmers in the district, in common with farmers in England and Wales, showed a small excess of mortality for stomach cancer in relation to the others in their social class ; hence the excess of mortality for Social Class II in relation to its population. The finding of an almost equally high rate for cancer of the intestine was unexpected in the light of the normal standardised mortality ratios which have been recorded for this site in Wales. The standardised mortality ratios for this site in Llanfyllin R.D. were 142·9 (male) and 149·8 (female).

The other four districts, with the exception of the relatively urban areas, were also found to have high gastric cancer mortality ratios. In one of the boroughs however, there was a suggestion that most of the stomach cancer deaths occurred at strictly rural residences, although otherwise this town showed a representation of population, social class and stomach cancer mortality not unlike that for England and Wales.

The discovery of a common dietary factor is not easy. Recent experimental work justifies alertness to the possibilities of water contamination and in the Llanfyllin R.D. much of the drinking water is virtually untreated though passing the necessary bacteriological standards ; furthermore it is derived from poorly drained peaty soils. Knowing that the Silurian formation of Mid-Wales possesses a significant degree of radioactivity, especially in certain shale beds, the public water supplies and many private supplies from houses where there had been a stomach cancer death were tested quantitatively for uranium oxide. The results showed no correlation with the cancer residences and their average, which was $0{\cdot}7 \times 10^{-6}$ g. per litre, was not considered to be higher than would be expected in most natural waters.

The possible significance of the uranium daughter elements is discussed in the light of the knowledge gained from studies of the fall-out of fission products. In 1956–57 the deposition of strontium-90 was 2600 $\mu\mu c$ per square metre at Milford Haven compared with 2260 $\mu\mu c$ per square metre for lead-210 which is the chief daughter element involved in natural fall-out. The alpha activities of these elements and the strontium-90 beta activities of certain foodstuffs were also found to be rather similar. As the dominant mode of contamination is by foliar retention and as samples of grass matt in Wales have been found to contain polonium-210 in amounts many times greater than those found in South East England, it is suggested that this element may gain access to the human body disproportionately through beef and mutton. These foodstuffs are likely to be derived from hill cattle and sheep which have grazed widely upon upland pastures of poor quality and in the area of the present study local slaughtering has always been the custom. Detailed measurements of the radioactivity of water supplies like those being made at present in West Devon, but also including meat supplies, should yield further information of value.

The gene frequencies of Montgomeryshire were not considered to have any bearing upon the incidence of stomach cancer.

I wish to express gratitude to those who helped me in this investigation: to Mr. S. H. U. Bowie, the Chief Geologist of the Geological Survey who undertook to provide the uranium oxide analyses; to the Institute of Cancer Research whose statistics on Sweden and other countries were made available; to my colleagues and members of my staff.

### REFERENCES

ABBATT, J. D., LAKEY, J. R. A. AND MATHIAS, D. J.—(1960) *Lancet*, ii, 1272.

AGRICULTURAL RESEARCH COUNCIL.—(1958) *Radiobiol. Lab. Rep.* No. 1.—(1959) *Ibid.*, No. 2.

AIRD, I., BENTHALL, H. H. AND ROBERTS, J. A. F.—(1953) *Brit. med. J.*, i, 799.

BERENBLUM, I. AND SHUBIK, P.—(1949) *Brit. J. Cancer*, **3**, 109.

BORNEFF, J.—(1960) *Bull. Hyg.*, **11**, 1077.

BRYANT, F. J., CHAMBERLAIN, A. C., MORGAN, A. AND SPICER, G. S.—(1958*a*) *Atomic Energy Res. Estab.*, HP/R 2056.—(1958*b*) *Ibid.*, HP/R 2353.

*Idem*, MORGAN, A. AND SPICER, G. S.—(1958) *Ibid.*, HP/R 2730.

BURTON, W. M. AND STEWART, N. G.—(1960) *Nature, Lond.*, **186**, 584.

COOK, J. W.—(1957) *Lancet*, i, 333.

DAVIES, R. I. AND GRIFFITH, G. W.—(1954) *Brit. J. Cancer*, **8**, 56.

DOBSON, G. M. B.—(1956) *Proc. Roy. Soc.*, **236**, 187.

EVANS, R.—(1959) *Rep. Minist. Hlth*, Carmarthenshire, p. 18.

FINK, R. M.—(1950) 'Biological Studies with Polonium, Radium, and Plutonium.' New York, London (McGraw-Hill).

GENERAL REGISTER OFFICE.—(1957) 'Studies on Medical and Population Subjects,' No. **13**, pp. 5, 23.

*Idem.*—(1951) Census. County Report (Merionethshire, Montgomeryshire, Radnorshire), pp. 70, 77.

HILL, C. R.—(1960) *Nature, Lond.*, **187**, 221.

HOWE, G. M.—(1959) *Brit. J. prev. soc. Med.*, **13**, 204.

JAKI, S. L. AND HESS, V. F.—(1958) *J. geophys. Res.*, **63**, 373.

JENNINGS, D., BALME, R. H. AND RICHARDSON, J. E.—(1956) *Lancet*, ii, 11.

LEGON, C. D.—(1952) *Brit. med. J.*, ii, 700.

MAYNEORD, W. V.—(1959) *Roy. Soc. Hlth J.*, **79**, 338.

*Idem*, TURNER, R. C. AND RADLEY, J. M.—(1960) *Nature, Lond.*, **187**, 208.

NICHOLSON, G.—(1960) *Publ. Hlth, Lond.*, **74**, 403.

OSTLE, D.—(1954) *Min. Mag.*, **91**, 201.

PEIRSON, D. H., CROOKS, R. N. AND FISHER, E. M. R.—(1960) *Atomic Energy Res. Estab.*, R.3358.

PONSFORD, D. R. A.—(1955) *Bull. geol. Surv. G.B.*, No. 10, p. 24.

REGISTRAR GENERAL.—(1951) *Decennial Supplement. England and Wales*, Occupational Mortality, Part I.—(1953) *Statistical Review of England and Wales*, Text Volume (Appendix B), 247.—(1954) *Ibid.*, Commentary, p. 135.—(1958) *Ibid.*, Commentary, pp. 127, 129, 131.

SIDGWICK, N. V.—(1950) 'The Chemical Elements and Their Compounds.' London (Oxford University Press).

SMITHERS, D. W.—(1959) *Lancet*, i, 589.

STEWART, N. G., OSMOND, R. G. D., CROOKS, R. N. AND FISHER, E. M. R.—(1957) *Atomic Energy Res. Estab.*, HP/R 2354.

*Idem*, CROOKS, R. N., OSMOND, R. G. D., OWERS, M. J. AND HEALY, C.—(1959) *Ibid.*, HP/R 2795.

STOCKS, P.—(1947) 'Regional and Local Differences in Cancer Death Rates.' London (H.M. Stationery Office).—(1958) *Rep. Brit. Emp. Cancer Campgn.* **35**, Supplement.—(1959) *Brit. med. J.*, i, 74.

*Idem* AND DAVIES, R. I.—(1960*a*) *Publ. Hlth. Lond.*, **74**, 408.—(1960*b*) *Brit. J. Cancer*, **14**, 8.

TROMP, S. W. AND DIEHL, J. C.—(1955) *Ibid.*, **9**, 349.

UNDERWOOD, E. J.—(1956) 'Trace Elements in Human and Animal Nutrition.' London (Academic Books).

WALTHER, W. W., RAEBURN, C. AND CASE, J.—(1956) *Lancet*, ii, 970.

WATKIN, I. M.—(1956) *Heredity*, **10**, 161.

WEDD, C. B., SMITH, B., KING, W. B. R. AND WRAY, D. A.—(1929) *Mem. geol. Surv. U.K.*, p. 52.